# Genome-wide screening and functional analysis identify a large number of long noncoding RNAs involved in the sexual reproduction of rice

**DOI:** 10.1186/s13059-014-0512-1

**Published:** 2014-12-03

**Authors:** Yu-Chan Zhang, Jian-You Liao, Ze-Yuan Li, Yang Yu, Jin-Ping Zhang, Quan-Feng Li, Liang-Hu Qu, Wen-Sheng Shu, Yue-Qin Chen

**Affiliations:** Key Laboratory of Gene Engineering of the Ministry of Education, State Key Laboratory for Biocontrol, School of Life Science, Sun Yat-Sen University, Guangzhou, 510275 PR China; Guangdong Provincial Key Laboratory of Malignant Tumor Epigenetics and Gene Regulation, Research Center of Medicine, Sun Yat-sen Memorial Hospital, Sun Yat-sen University, Guangzhou, 510120 PR China

## Abstract

**Background:**

Long noncoding RNAs (lncRNAs) play important roles in a wide range of biological processes in mammals and plants. However, the systematic examination of lncRNAs in plants lags behind that in mammals. Recently, lncRNAs have been identified in *Arabidopsis* and wheat; however, no systematic screening of potential lncRNAs has been reported for the rice genome.

**Results:**

In this study, we perform whole transcriptome strand-specific RNA sequencing (ssRNA-seq) of samples from rice anthers, pistils, and seeds 5 days after pollination and from shoots 14 days after germination. Using these data, together with 40 available rice RNA-seq datasets, we systematically analyze rice lncRNAs and definitively identify lncRNAs that are involved in the reproductive process. The results show that rice lncRNAs have some different characteristics compared to those of *Arabidopsis* and mammals and are expressed in a highly tissue-specific or stage-specific manner. We further verify the functions of a set of lncRNAs that are preferentially expressed in reproductive stages and identify several lncRNAs as competing endogenous RNAs (ceRNAs), which sequester miR160 or miR164 in a type of target mimicry. More importantly, one lncRNA, XLOC_057324, is demonstrated to play a role in panicle development and fertility. We also develop a source of rice lncRNA-associated insertional mutants.

**Conclusions:**

Genome-wide screening and functional analysis enabled the identification of a set of lncRNAs that are involved in the sexual reproduction of rice. The results also provide a source of lncRNAs and associated insertional mutants in rice.

**Electronic supplementary material:**

The online version of this article (doi:10.1186/s13059-014-0512-1) contains supplementary material, which is available to authorized users.

## Background

Non-protein-coding RNAs (ncRNAs) constitute a substantial portion of transcribed sequences with structural, regulatory or unknown functions. Because of these important biological roles, ncRNAs have been of great research interest in recent years. Attention was previously given to small regulatory RNAs (sRNAs), such as microRNAs (miRNAs), which are less than 200 nucleotides in length [1]. ncRNAs longer than 200 nucleotides (long non-coding RNAs (lncRNAs)) were found to have functions associated with virtually every biological process in mammals, and these initial reports initiated a wave of research on lncRNAs that followed the path of sRNA research. Recently, lncRNAs have emerged as potent regulators, particularly in mammals. However, studies on lncRNAs in plants remain at the early stage; only a few lncRNAs have been shown to regulate plant development, especially during reproduction [2].

Sexual reproduction is one of the most essential biological processes and occurs in a vast number of species. Numerous studies have been devoted to the identification of reproduction-related genes, making great progress in understanding the reproductive processes of both animals and plants. However, the complex regulatory networks involving these genes remain largely unknown. Intriguingly, many lncRNAs have recently been proven to play important roles in reproductive processes through the regulation of related genes in various species. In mammals, lncRNAs, such as Xist, H19, Kcnq1ot1, bxd and HOTAIR, have been found to be crucial for the precise control of embryogenesis [3-6]. Notably, several plant lncRNAs have also been demonstrated to participate in reproductive regulation, including COLDAIR, COOLAIR, LDMAR, CsM10 and Zm401 [7-11], indicating that one of the principal functions of plant lncRNAs might be to regulate plant reproduction. More interestingly, Komiya *et al*. [12] found that a number of large intergenic non-coding RNAs (lincRNAs) could generate 21-nucleotide phasiRNAs, which associate with the germline-specific Argonaute (AGO) proteinMEL1 in rice, indicating that rice lncRNAs might play a role in the development of pre-meiotic germ cells. Genome-wide analysis is necessary to discover new lncRNAs and is important for the further functional analysis of these RNAs. More than 8,000 lncRNAs have been identified in humans using bioinformatic methods [13], and approximately 4,000 lncRNAs have been identified in mice [14,15]. In plants, 6,480 transcripts have been classified as lncRNAs in *Arabidopsis* [16,17], and 125 putative stress-responsive lncRNAs have been identified in wheat [18]. Although rice is a model species for plant development studies and represents a staple food for nearly half of the global population, rice lncRNAs remain poorly characterized, and no systematic screening of potential lncRNAs in the rice genome has been reported.

In this study, we performed whole transcriptome strand-specific RNA sequencing (ssRNA-seq) of samples obtained from rice anthers, pistils, seeds that were harvested 5 days after pollination (DAP) and shoots that were harvested 14 days after germination (DAG). Together with 40 available rice RNA-seq datasets, we systematically identified rice lncRNAs (including lincRNAs and antisense lncRNAs) with a specific focus on the lncRNAs that were expressed at reproductive stages and performed functional studies on some of these reproduction related lncRNAs. Our results indicated that a number of rice lncRNAs are highly tissue-specific, and a large portion of these RNAs are specifically expressed in conjunction with reproduction-related processes, particularly in pollen.

## Results

### A computational approach for the genome-wide identification of lncRNAs in rice

**Figure 1:**
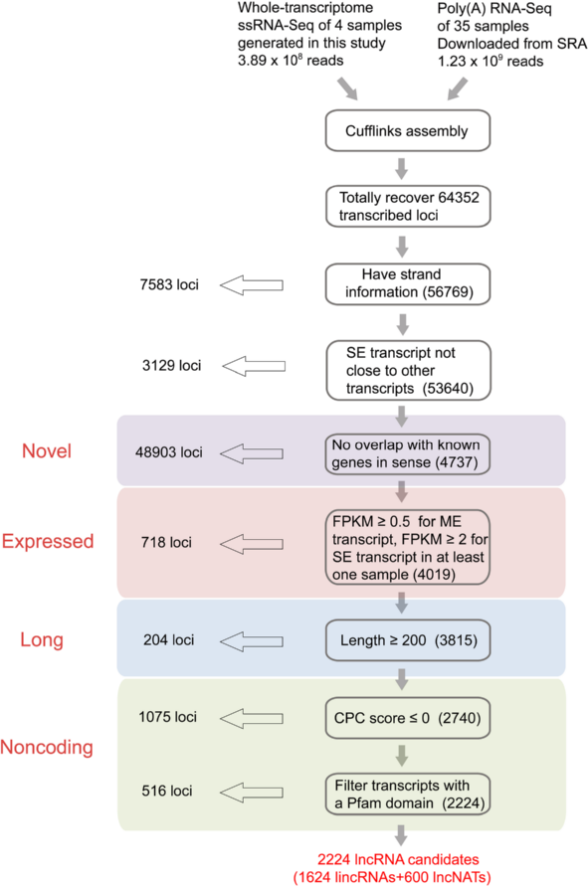
**An integrative computational pipeline for the systematic identification of lncRNAs in rice.** CPC,Coding Potential Calculator; lncNAT, long non-coding natural antisense transcript; ME, multiple exon; SE, single exon.

To systematically identify lncRNAs related to rice reproduction, we performed whole transcriptome ssRNA-seq of rice anthers, pistils, seeds that were harvested 5 DAP and shoots that were harvested 14 DAG (the sequencing results included 3.89 × 10^8^ reads; Additional file 1; Sequence Read Archive (SRA) accession number SRP047482). We then developed a rice lncRNA computational identification pipeline based on RNA-seq data (Figure 1) using 4 whole transcriptome ssRNA-seq data sets and 40 available poly(A) RNA-seq data sets (1.23 × 10^9^ reads). These datasets covered most of the organs and stages involved in rice reproduction (Additional file 1) and were suitable for the identification of reproduction-related lncRNAs. Our lncRNA identification strategy comprised three key procedures (Figure 1). First, the rice transcriptome was reconstructed from all of the RNA-seq datasets using Cufflink 2.0 [19]. After filtering out infrequently expressed transcripts (those showing FPKM (fragments per kilobase of transcript per million mapped reads) scores <0.5 in all samples) and transcripts without strand information, we recovered 77.4% (30,219/39,045) of the non-transposableelement (non-TE)-related mRNAs in the datasets (the mRNAs discussed in the following sections are non-TE-related mRNAs unless otherwise specified). The efficient recovery of known protein-coding genes indicated that the dataset employed here was suitable for the recovery of novel transcribed regions of the rice genome.

Second, we only retained novel (not overlapping with known genes in sense), large (longer than 200 nucleotides), expressed (for multiple-exon transcripts FPKM ≥0.5, for single-exon transcripts FPKM ≥2) transcripts. All single-exon transcripts close to other transcripts were removed. We then evaluated the coding potential of the remaining transcripts and obtained novel expressed lncRNAs. We used the Coding Potential Calculator (CPC) [20] to predict the coding potential of each transcript. All transcripts with CPC scores >0 were discarded. To guarantee the thorough elimination of protein-coding transcripts, we also employed HMMER [21] to scan each transcript with a CPC score <0 in all three reading frames to exclude transcripts that encoded any of the known protein domains cataloged in the Pfam protein family database [22]. Finally, we obtained 2,224 reliably expressed lncRNAs (Additional file 2), including 1,624 lincRNAs and 600 long non-coding natural antisense transcripts (lncNATs), which intersect any exon of a protein-coding mRNA on the opposite strand.

### The genomic characteristics and conservation of rice lncRNAs

**Figure 2:**
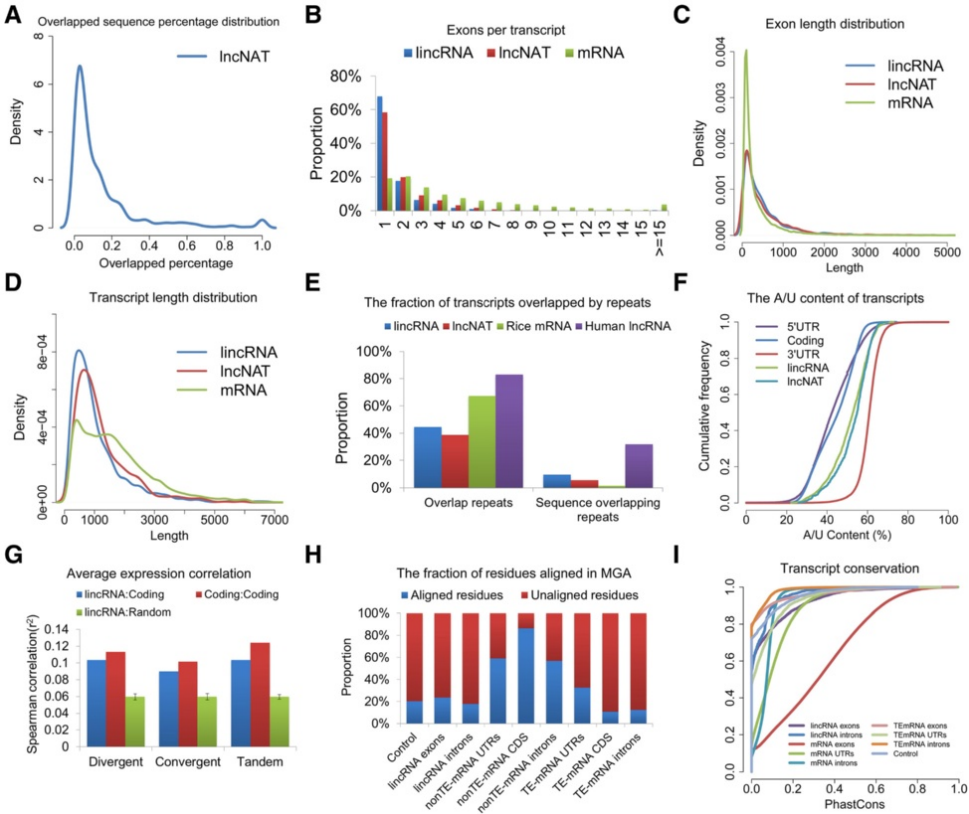
**Properties of rice lncRNAs. (A)** The proportion of lncNAT sequences overlapped by mRNAs. **(B)** The number of exons per transcript for all lincRNAs and lncNATs and protein-coding transcripts. **(C) **Exon size distributions for lincRNAs, lncNATs and protein-coding transcripts. **(D)**Transcript size distributions for lincRNAs, lncNATs and protein-coding transcripts. **(E)** The fraction of transcripts overlapping at least one base of a repetitive element (left), and the fraction of all transcript sequences overlapping repeats (right). The repeat elements found in rice were annotated using RepeatMasker [28], whereas the repeat elements found in human sequences were downloaded from the UCSC genome browser [29] and were also annotated by RepeatMasker. **(F) **A/U content of rice lincRNAs and lncNATs and various regions of protein-coding transcripts. **(G) **Correlation between the expression levels of lincRNAs and the expression levels of their closest protein-coding genes. The Spearman correlation between the expression levels of each gene across 17 stages/tissues and the expression levels of the closest protein-coding gene were calculated. Average values are shown in the plot. Error bars represent standard deviations based on 1,000 random shuffles of lincRNA positions. **(H)** The fraction of residues aligned in multiple-genome alignments (MGA) for the indicated protein-coding gene, TE-mRNA and lincRNA regions. The controls are random intergenic regions that were size- and-chromosome matched to the lincRNA set.CDS, coding sequence. **(I) **The level of conservation of the exons, introns and UTRs (if any) of protein-coding genes, TE-mRNAs and lincRNAs. The cumulative distributions of mean phastCons scores derived from eight-way whole-genome alignments are shown.

We characterized the basic genomic features of the obtained lncRNAs and compared these features with the available features of *Arabidopsis* or human lncRNAs or to rice protein-coding genes where appropriate. We found that only a small fraction (median percentage, 6.5%) of the sequence of most of the lncNATs was antisense overlapped by protein-coding mRNA (Figure 2A) and that lincRNAs and lncNATs are similar in many aspects (Figure 2). To display the characteristics of lincRNAs and lncNATs more clearly, we analyzed the characteristics of lincRNAs and lncNATs separately in the following comparisons. Similar to findings for *Arabidopsis* [16,23], only around half of lncRNAs were spliced (46.5% for lincRNAs, 65.9% for lncNATs). In contrast, more than 98% of human lncRNAs are spliced [24] (Figure 2B). Rice lncRNAs have fewer exons than mRNAs (2.21 versus 4.67 on average, respectively; 2.10 exons for lincRNAs and 2.42 exons for lncNATs), but their exon lengths (median length of 323 nucleotides; 322 nucleotides for lincRNAs, 298 nucleotides for lncNATs) are longer than those of mRNA (median length of 159 nucleotides) (Figure 2C). Full-length rice lncRNA transcripts (median length of852 nucleotides; 800 nucleotides for lincRNAs, 950 nucleotides for lncNATs) are longer than *Arabidoposis* lncRNA transcripts (median length of 285 nucleotides) [16,23] and human lncRNA transcripts (median length of 592 nucleotides) [24], and are generally shorter than protein-coding transcripts (median length of 1,411 nucleotides) (Figure 2D). Rice lncRNAs generally do not overlap with repeat sequences (Figure 2E); fewer repeats-overlapped rice lncRNAs than repeats-overlapped rice mRNAs and repeats-overlapped human lncRNAs. Like *Arabidopsis* lncRNAs [16,23], only a small proportion of rice lncRNAs (122 of 1,624 lincRNAs, 7.5%; 44 of 600 lncNATs, 7.3%) generate sRNAs (Additional file 3), implying that these lncRNAs might function through generating sRNAs. Interestingly, rice lncRNAs were much more A/U-rich than the coding sequences and the 5′UTRs of protein-coding genes but were less A/U-rich than 3′UTRs that use A/U-rich elements to regulate mRNA degradation [25] (Figure 2F). This characteristic is conserved in *Arabidopsis* (Figure S1A in Additional file 4) and animal lncRNAs [26,27], implying that this feature might be related to the functions of lncRNAs. Rice lincRNAs are most likely to appear in divergent orientations with respect to the closest neighboring protein-coding genes (Figure S1B in Additional file 4). However, we did not observe a stronger correlation between the expression of rice lincRNAs and their nearest neighbors than that between adjacent protein-coding genes (Figure 2G), although the expression of lncNATs is more highly correlated with convergent and divergent overlapped mRNA than with tandem overlapped mRNAs (Figure S1C in Additional file 4).

We further analyzed the conservation of rice lncRNAs through an eight-way genomic alignment between the genomes of rice [30], *Musa* [31], *Arabidopsis* [32], *Brachypodium* [33], maize [34], poplar [35], grapevine [36] and *Sorghum* [37] using MultiZ [38]. Our analysis mainly focused on lincRNAs because the partial antisense overlap of lncNATs might have interfered with the analysis. The genomes were then aligned to determine the conservation score (consScore) of each nucleotide in the rice genome using phastCons [39]. The fraction of lincRNA residues that aligned in the whole-genome alignments was 23.5%; this value is much higher than those of TE-mRNA coding sequences (10.7%) and TE-mRNA introns (12.3%) but is much smaller than those of mRNA coding sequences (86.3%), UTRs (59.1%) and introns (56.9%) and is comparable to those of lincRNA introns (18.0%) and intergenic controls (20.2%) (Figure 2H). We further measured the conservation of lincRNAs based on the obtained consScores. The exons of lincRNAs were more conserved than the introns of lincRNAs and control exons with matched lengths (Figure 2I). Interestingly, both the exons and introns of the studied lincRNAs were more conserved than those of TE-related mRNAs. However, the rice lincRNAs were less conserved than mRNA introns, possibly due to the presence of conserved ncRNAs (such as small nucleolar RNAs (snoRNAs)) in mRNA introns [40]. Previous studies have shown that both plant and animal lncRNAs can function through short conserved regions (despite the rapid sequence evolution observed elsewhere in these RNAs) and have indicated that lincRNAs in animals are likely to contain short conserved regions [26,27,41,42]. However, we found that rice lincRNAs do not contain shorter conserved regions than protein-coding genes (Figure S1D in Additional file 4), suggesting that lincRNAs that function through short conserved regions might not be as common in rice as in animals.

### Rice lncRNAs are highly tissue-specific, and many lncRNAs are specifically expressed during reproduction

**Figure 3:**
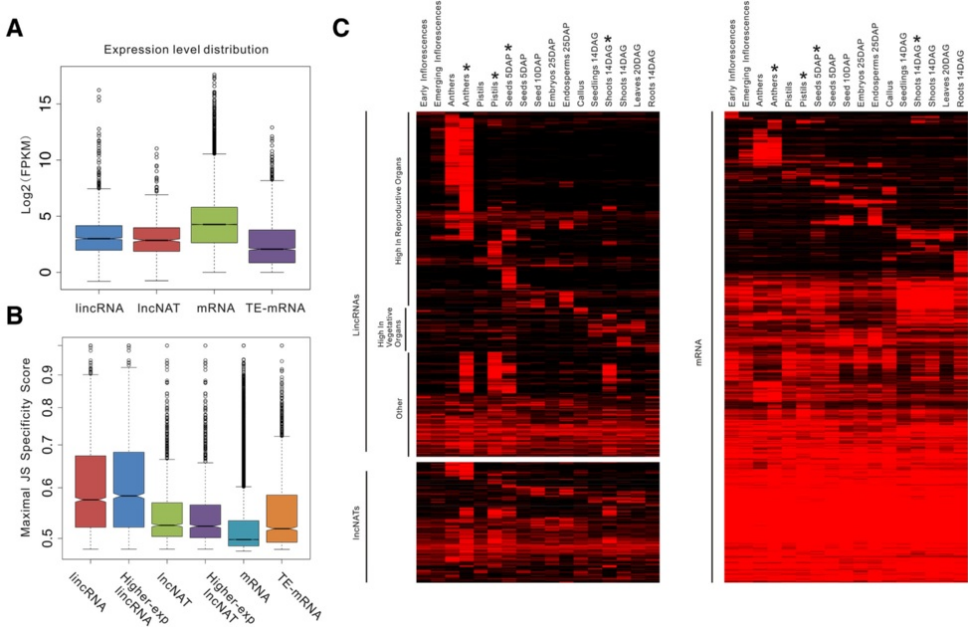
**Developmental and tissue-specific expression of lncRNAs. (A) **lincRNAs and lncNATs are expressed at lower levels than protein-coding genes but at higher levels than TE-mRNAs. FPKM, fragments per kilobase of exons per million fragments mapped. **(B)** The maximal JS (Jensen-Shannon)specificity score distributions for all lincRNAs, highly expressed lincRNAs, lncNATs, highly expressed lncNATs, protein-coding genes and TE-mRNAs. **(C)** The abundance of all of the expressed lincRNAs and lncNATs (left panel) and protein-coding genes (right panel) across 17 stages/tissues. The rows and columns were ordered according to CLICK. The asterisks marked the four strand-specific whole transcriptome sequencing datasets.

We then estimated the expression level of each transcript using FPKM and found that the lincRNAs and lncNATs were expressed at similar levels (median: 8.0 FPKM versus 7.21 FPKM, respectively), which were lower than the levels at which protein-coding genes are expressed (median: 19.3 FPKM, both *P* < 2.2 × 10^−16^, *t*-test) but higher than the levels at which TE-related mRNAs are expressed (median: 4.2 FPKM, both *P* < 2.2 × 10^−16^, *t*-test) (Figure 3A). We further estimated the degree of the differential expression of lincRNAs, lncNATs, mRNAs and TE-related mRNAs based on the JS (Jensen-Shannon) score [13]. Intriguingly, we found that lincRNAs tend to be far more differentially expressed than lncNATs (*P* < 2.2 × 10^−16^, Kolmogorov-Smirnovtest), which exhibited a similar degree of differentiated expression to that of TE-mRNAs; both lincRNAs and lncNATs are more differentially expressed than mRNAs (both *P* < 2.2 × 10^−16^, Kolmogorov-Smirnovtest) (Figure 3B). The lower expression level and highly differentiated expression pattern of lincRNAs were also found in *Arabidopsis* and animals [13,16], suggesting that both of these characteristics are conserved for lincRNAs.

The highly tissue-specific expression pattern observed for lincRNAs suggests that it might be possible to classify lincRNAs according to their expression patterns. We clustered the lincRNAs based on their expression patterns in 13 different types of tissue samples using CLICK [43]. Remarkably, the lncRNAs can be classified into three categories: lincRNAs that are highly expressed in reproductive organs (including panicles, anthers, pistils, seeds 5DAP, seeds 10DAP, embryos 25DAP and endosperms 25DAP); lincRNAs that are highly expressed in vegetative organs (including callus, seedlings 14DAG, shoots 14DAG, leaves 20DAG and roots 14DAG), and other lincRNAs (lincRNAs expressed in multiple organs or only expressed in our sequencing datasets); lncNATs were separately categorized (Figure 3C; Additional file 2).

**Figure 4:**
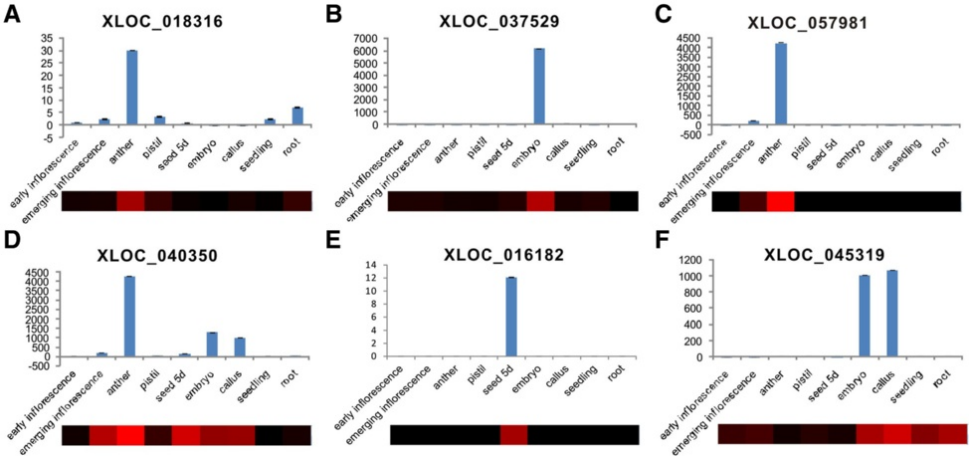
**Confirmation of the expression patterns of lncRNAs using quantitative RT-PCR. (A-F)** The expression pattern of four multi-exonic lncRNAs **(A-D)** and two single-exon lncRNAs **(E,F)**. A heatmap of each lncRNA was generated from the FPKM values and used to visualize the lncRNA expression patterns in the RNA-seq data. The values shown are the means ± standard deviation of n =3 replicates. Actin2 was used as the reference gene.

**Figure 5:**
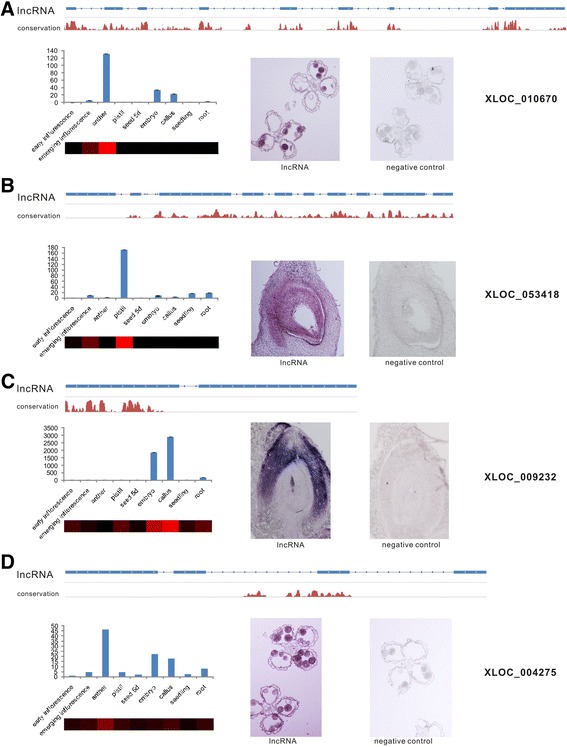
**Expression pattern and spatial expression pattern analysis of four conserved lncRNAs based on qRT-PCR and** ***in situ*** **hybridization. (A-D)** The top part of each panel shows the conservation of a particular lncRNA; the lower left of each panel shows the expression pattern of the lncRNA based on qRT-PCR, and the lower right of each panel shows the spatial expression pattern in sperm **(A,D)** coleoptile **(B)** or ovule **(C)** of the particular lncRNA examined by *in situ* hybridization. The antisense transcript of each lncRNA was used to detect the expression of corresponding lncRNAs by *in situ* hybridization, and the antisense transcripts of lncRNAs that were not expressed in the corresponding organs were used as a negative control. A heatmap of each lncRNA was generated from the FPKM values and was used to visualize the lncRNA expression patterns in the RNA-seq data. The values shown are the means ± standard deviation (n =3 replicates). Actin2 was used as the reference gene.

Interestingly, we found that a number of rice lncRNAs are specifically expressed at a single development stage, and this type of lncRNA was expressed during the integrated sexual reproduction process (Figure 3C; Additional file 2), indicating that lncRNAs may function throughout the entire reproductive process in rice. To confirm the expression patterns of the lncRNAs, we randomly selected 10 lncRNAs, including both single-exon lncRNAs (XLOC_045319, XLOC_016182) and multi-exonic lncRNAs (XLOC_018316, XLOC_037529, XLOC_057981, XLOC_040350, XLOC_010670, XLOC_009232, XLOC_053418 and XLOC_004275) (Figures 4 and 5), and validated their expression patterns using real-time quantitative PCR (qRT-PCR). We found a nearly perfect concordance between our experimental results and the RNA-seq results for most of the studied tissues, suggesting that the lncRNA expression patterns based on RNA-seq data are reliable.

We also performed *in situ* hybridization to analyze the spatial expression patterns of 4 of these 10 validated lncRNAs that were preferentially expressed during reproductive stages and that exhibited high abundance and conservation (Figure 5). Interestingly, these lncRNAs exhibited higher degrees of tissue specificity in their expression patterns than we observed in their expression profiles, and their expression tended to be restricted to particular types of cells - for example, XLOC_010670 and XLOC_004275 are highly expressed in sperm (Figure 5A,D); XLOC_053418 is specifically expressed in ovules (Figure 5B); and XLOC_009232 is specifically expressed in coleoptiles (Figure 5C) - rather than being ubiquitously expressed throughout the tissue.

Of the identified reproduction-related lincRNAs, most were specifically expressed in anther (58.0%) (Figure 3C). This finding is similar to previous observations obtained in animals: a large proportion of lincRNAs are specifically expressed in the testis [13]. However, this phenomenon was not apparent for rice lncNATs. Thus, this characteristic might be specific for lincRNAs, which suggests that lincRNAs play important and conserved roles in male gametophyte genesis and in the regulation of reproductive growth. To further investigate whether the lincRNAs that are specifically expressed during reproduction have corresponding functions, we performed gene ontology (GO) analyses of mRNAs for which the expression patterns were correlated with lincRNAs that were highly expressed in reproductive organs and those that were correlated with lincRNAs that were highly expressed in vegetative organs. We found that the mRNA group exhibiting expression patterns correlated with ‘reproductive lincRNAs’ were significantly enriched in reproduction-specific GO terms, whereas the mRNA group correlated with ‘vegetative lincRNAs’ did not show such enrichment (Figure 3D; Additional file 5), indicating that lincRNAs that are specifically expressed during the reproductive process might function in regulating reproductive growth.

### Insertional mutant analysis reveals a set of lincRNAs that participate in reproduction

The results described above suggest that a set of lncRNAs might be associated with the regulation of reproduction. To investigate the functions of rice lncRNAs in the regulation of reproduction, we first performed a preliminary functional analysis of all of the identified lincRNAs in association with all nine existing rice mutant databases, including the affjp [44,45], cirad [46,47], gsnu, ostid [48], pfg [49], rmd [50], ship, trim and ucd databases (Additional file 6). lncNATs were not selected for mutant analysis because they are partially overlapped with protein-coding genes, which might produce false positive results. Our strategy was to blast the flanking sequence tags (FSTs) included in each mutant database against the 1,624 lincRNAs and their 1-kb upstream regions separately. A total of 736 lincRNA-related insertional mutants were found in these databases (Additional file 7). Among these mutants, 233 lincRNAs were related to mutants with insertions in their transcribed regions, and 227 were related to mutants with insertions in their potential promoter regions, as determined from at least one mutant database (Table 1). These mutants would contribute to the prospective functional analysis of individual lincRNAs.

**Table 1:** **Numbers of available lincRNA-correlated rice mutants**

**Mutant database** ^**a**^	**Internal**			**Upstream**		
	**Mutant number**	**lncRNA number**	**With phenotype information**	**Mutant number**	**lncRNA number**	**With phenotype information**
affjp	47	30	27	37	19	14
cirad	178	117	73	209	131	90
gsnu	3	3	-	6	4	-
ostid	2	2	-	1	1	-
pfg	29	30	-	12	12	-
rmd	16	13	-	8	7	-
ship	3	3	-	2	1	-
trim	76	62	-	72	59	-
ucd	13	11	-	22	12	-

^a^Detailed information on each mutant database can be found in Additional file 6: Table S4.

Of the nine available rice mutant databases, only affjp includes published phenotypic data [45], and this database was therefore selected for use in a further analysis of the relationships between the expression patterns of the lincRNAs and their phenotypes. Among the 84 mutants found in the affjp database, 47 exhibited Tos17 insertions in the transcribed regions of 30 lincRNAs, whereas 37 showed insertions in the potential promoter regions of 19 lincRNAs. Moreover, 76.7% of the lincRNAs with insertions in their transcribed regions and 73.7% of the lincRNAs with insertions in their promoter regions are associated with observed phenotypes. The phenotypes related to these lincRNAs have been summarized and are presented with their expression patterns to allow readers to search for lincRNAs of interest in Additional file 8.

**Figure 6:**
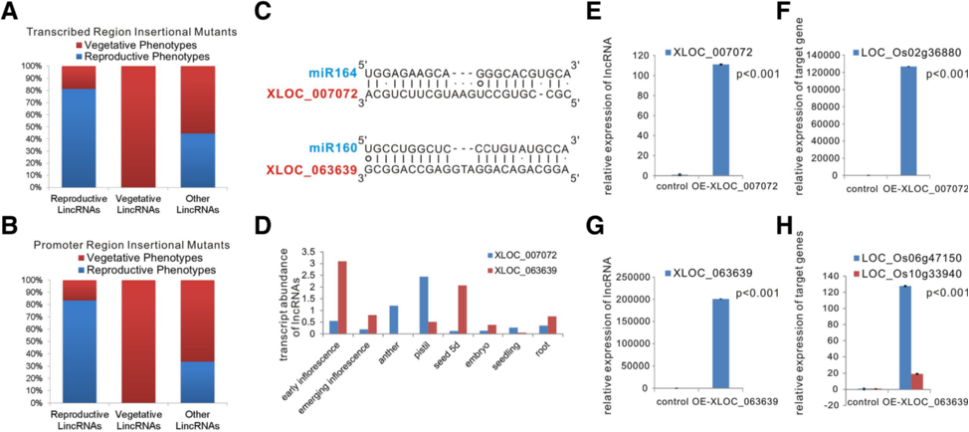
**Functional analysis of rice lncRNAs. (A,B)** The proportion of lncRNAs that are highly expressed during reproductive or vegetative stages or with unbiased expression patterns that display phenotypes that are related to reproductive growth or vegetative growth in their transcribed region insertional mutants **(A)** and promoter region insertional mutants **(B)**. **(C-H)** Functional analysis of two miRNA ‘decoy’ lncRNAs. **(C)** Predicted base-pairing interaction of OsmiR164-XLOC_007072 and OsmiR160-XLOC_063639. **(D)** Transcript abundance of XLOC_007072 and XLOC_063639. **(E)** Detection of XLOC_007072 expression in the control vector and in the XLOC_007072-overexpression vector in transiently transformed rice protoplasts using qRT-PCR. **(F)** qRT-PCR analysis of the OsmiR164 target gene in the control vector and in the XLOC_007072-overexpression vector in transiently transformed rice protoplasts. **(G)** Detection of XLOC_063639 expression in the control vector and in the XLOC_063639-overexpression vector in transiently transformed rice protoplasts using qRT-PCR. **(H)** qRT-PCR analysis of OsmiR160 target genes in the control vector and in the XLOC_007072-overexpression vector in transiently transformed rice protoplasts. The values shown in **(E**-**H)** are means ± standard deviation. Actin2 was used as the reference gene (n =3 replicates). Significant differences were identified at the 1% probability level using Student’s *t*-test.

We divided the observed phenotypes into two groups: phenotypes related to reproductive growth, such as low fertility, sterility, abnormal panicles and heading dates; and phenotypes related to vegetative growth, including height, tillering, lethality, germination and leaf-related phenotypes. Of these mutants, 45.9% possess phenotypes related to reproductive growth for the transcribed region insertional mutants, and 45.0% possess phenotypes related to reproductive growth for the promoter region insertional mutants (Additional file 8). Intriguingly, more than 80% of the lincRNAs that were highly expressed in reproductive organs caused phenotypes related to reproductive growth with insertions in either transcribed regions or promoter regions, and only 30 to 40% of the lincRNAs that belong to the ‘other’ group (with unbiased expression patterns) caused phenotypes related to reproductive growth (Figure 6A,B). This finding provides further support for the notion that lincRNAs that are preferentially expressed at the reproductive stage regulate reproductive growth.

### Functional analysis of several reproduction-related lncRNAs elucidated their roles as competing endogenous RNAs or participants in the regulation of reproduction

It has been shown that lncRNAs function as competing endogenous RNAs (ceRNAs) by binding to and sequestering specific miRNAs in a type of target mimicry to protect the target mRNAs from repression in both plants and animals [41,42,51-57]. Because many miRNAs have been reported to regulate reproduction in plants [58], we predicted lncRNAs that might act as ceRNAs using the algorithm developed by Wu *et al*. [42]. Interestingly, 65 of the identified rice lincRNAs were predicted to be ‘decoys’ of conserved miRNAs, such as miR160, miR164, miR168, miR169 and miR408 (Additional files 9 and 10). We further used a transient transformation assay to test whether these lncRNAs could function as miRNA decoys. Expression vectors under the control of the 35S promoter containing a decoy lncRNA (XLOC_0063639 or XLOC_007072) that is highly expressed during the reproductive stage were introduced into rice protoplasts separately (see the Materials and methods section for details). Twenty-four hours after transformation, the total RNA of the protoplasts was extracted, and the relative expression level of the lncRNAs and the endogenous target genes of the corresponding miRNAs were measured by qRT-PCR. Both XLOC_0063639 and XLOC_007072 dramatically increased the mRNA abundance of corresponding miRNA (OsmiR160 and OsmiR164) targets (LOC_Os02g36880 for miR164 [59]; LOC_Os06G47150 and LOC_Os10g33940 for miR160 [60]) in their transiently expressed protoplasts, suggesting that XLOC_0063639 and XLOC_007072 indeed inhibited the functions of OsmiR160 and miR164, respectively (Figure 6C,E-H). It is known that OsmiR160 and OsmiR164 participate in regulating floral and seed development in plants [61-63]; interestingly, XLOC_007072 is specifically expressed in pistil and anther, and XLOC_0063639 is highly expressed in early panicles and seeds after pollination (Figure 6D). Thus, these two miRNA-lncRNA functional pairs might be important regulators of floral and/or seed development. Further studies are necessary to investigate the functions of these two lncRNAs in sequestering miRNAs *in vivo*.

**Figure 7:**
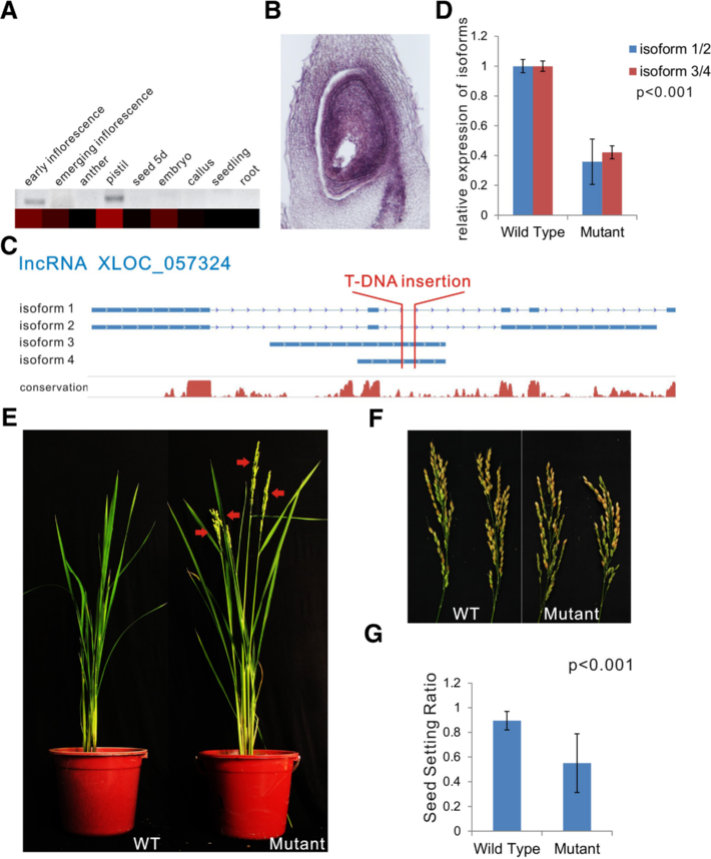
**Functional analysis of lncRNA XLOC_057324. (A)** Confirmation of the expression pattern of XLOC_057324 using semi-quantitative PCR. A heatmap was generated from the FPKM values and was used to visualize the lncRNA expression pattern in the RNA-seq data. **(B)** Spatial expression pattern analysis of XLOC_057324 based on *in situ* hybridization. **(C) **Isoforms and conservation of XLOC_057324; the T-DNA insertional site of the mutant analyzed in this study is marked in red. **(D)** Relative expression levels of XLOC_057324 isoforms in the mutant plants detected using qRT-PCR. **(E) **Phenotypes of wild-type (WT) plants and mutant plants during flowering; panicles are marked using red arrows. **(F,G) **Panicles **(F)** and seed setting ratios **(G)** of wild-type and mutant plants after harvest. The values shown in **(D,G)** are means ± standard deviation;n =3 replicates in **(D)**; n =20 plants in **(G)**. Actin2 was used as the reference gene. Significant differences were identified at the 1% probability level using Student’s *t*-test.

We also studied a lncRNA (XLOC_057324) that is highly expressed in reproductive organs in relation to its physiological function in rice plants. First, we confirmed the expression pattern of this lncRNA using qRT-PCR and *in situ* hybridization. The results showed that this lncRNA is specifically expressed in young panicles and pistils (expression was restricted to ovules), suggesting that XLOC_057324 might play a role in regulating panicle and/or pistil development (Figure 7A,B). A rice mutant from the rmd database [50] that contains a T-DNA insertion in the lncRNA, XLOC_057324, was used for further functional analysis (Figure 7C). We first re-identified the T-DNA insertional site and then analyzed the expression of XLOC_057324 and the phenotypes caused by the insertion. Note that no gene is located 15 kb downstream of the T-DNA insertional site, and although one gene (LOC_Os08g35520.1) is located upstream of the insertional site, we did not detect any expression nor visible difference in the expression of this gene between wild-type plants and mutant plants (Figure S1E in Additional file 4). Thus, the mutant phenotypes are most likely to be caused by the effect of the insertion into the lncRNA XLOC_057324. This mutation apparently reduced the abundance of all isoforms of this lncRNA (Figure 7D). As the mutant plant was generated using the japonica rice varieties Zhonghua 11 (ZH11), we further compared the phenotypes of the mutant plants with the ZH11 wild-type plants. Interestingly, the T1 and T2 mutant plants all flowered earlier than the wild-type plants when these plants were grown at the same time (Figure 7E), but the fertility decreased significantly (Figure 7F,G), indicating that XLOC_057324 is involved in panicle development and sexual reproduction.

## Discussion

Sexual reproduction is a crucial step in the life cycle of plants and is dominant among angiosperms; sexual reproduction is more crucial for crop plants because of its applications in agriculture. Over the past decade, genetic screens have identified a number of genes involved in sexual reproduction; however, the regulatory pathways that mediate the specification of reproductive organs and the process of embryogenesis are far from being understood. The recent discovery of lncRNAs has filled gaps in our knowledge of certain reproductive regulatory pathways. Although an increasing number of reports indicate that lncRNAs function in the regulation of reproduction in mammals, the identification of such lncRNAs in plants was just beginning, and only few plant lncRNAs have been shown to play roles in regulating reproductive processes [7-10]. In this study, we systematically identified and analyzed rice lncRNAs to find novel lncRNAs associated with sexual reproduction. A source of lincRNA-associated mutants was also provided to facilitate further functional analyses of rice lincRNAs. Moreover, we identified that several lncRNAs that are highly expressed in reproductive organs function as ceRNAs or participate in rice flowering and fertility processes. A number of lncRNAs were found for the first time to be specifically expressed during the reproductive stage and involved in reproduction.

lncRNAs have previously been identified in several species [13-16,18]. Human lncRNAs and *Arabidopsis* lncRNAs were selected for comparison in this study. Only characteristics that have been previously analyzed in human lncRNAs or in *Arabidopsis* lncRNAs were compared. Because *Arabidopsis* lncRNAs were identified using an entirely different method (tilling array) [16], some differences between *Arabidopsis* lncRNAs and rice lncRNAs might be due to the different identification methods used; for example, the tilling array method cannot be used to determine lncRNA introns, which might lead to an inaccurate estimation of the number of spliced *Arabidopsis* lncRNAs. However, after discarding subtle differences (such as the fact that plants produce more single-exon lncRNAs than humans, rice lncRNAs are longer than lncRNAs in *Arabidopsis* and human, short conserved elements are absent in plants, and differences exist in the exon numbers of lncRNAs between these three species), it is interesting that the overall characteristics of rice lncRNAs are similar to those of lncRNAs in *Arabidopsis* and human. For example, all lncRNAs in these organisms are shorter than mRNAs, can be spliced, are enriched in A/U and are non-conserved in sequence, indicating that lncRNAs may represent a type of conserved genes in eukaryotes that are undergoing rapid sequence evolution [26].

In addition, lncRNAs might have similar regulatory mechanisms in both plants and animals to some extent. It has been reported that lncRNAs could regulate various stages of gene expression either in *cis* or in*trans* [64]. *Cis*-acting lncRNAs were first reported to control the expression of genes that are positioned in the vicinity of their transcription sites [65,66]. Soon afterwards, more and more *trans*-acting lncRNAs have also been discovered, which can regulate gene expression at independent loci [6,67-71]. In this study, we have found that rice lncRNAs are not more preferred to be coexpressed with their neighboring genes than protein coding genes. This phenomenon has also been shown in animals [13,26,72]. Our findings, together with previous reports in animals, suggest the dominant mechanism of lncRNAs might not occur in *cis*, especially lincRNAs function in both plants and animals.

It is generally considered that lncRNAs are highly tissue-specific in various species. This characteristic of lncRNAs might imply that they could function in maintaining tissue identity and in tissue development and differentiation. Interestingly, we found that rice lncRNAs are clearly enriched in anthers, similar to the finding that approximately one-third of human lincRNAs are specifically expressed in the testis, although their functions remain unclear [13]. This finding may hint at the importance of lncRNAs in male gametophyte genesis or in the regulation of reproductive growth. Komiya *et al*. [12] have previously reported that most MEL1-associated phasiRNAs are derived from lincRNAs that are specifically expressed in meiotic anthers and that contain miR2118 cleavage sites. MEL1, a rice AGO protein, has specific functions in the development of pre-meiotic germ cells and the progression of meiosis. This finding suggests that anther-specific lncRNAs might play roles in germ cell development or meiosis. In this study, we found that 58.0% of all of the identified rice lincRNAs are specifically expressed in anther; in addition, we identified many lncNATs in anthers, although the proportion is much less than that of lincRNAs. Thus, the enrichment of lincRNAs in the male reproductive organ suggests that lincRNAs have specific functions in male gametophyte genesis. We expect that more lincRNAs that are specifically expressed in male reproductive organs will be identified in other species in the future. The findings obtained in this study could therefore promote the functional analysis of rice lincRNAs.

In contrast to our understanding of small ncRNAs, little is known about the functions and regulatory mechanisms of lncRNAs. One intriguing mechanism is lncRNA-miRNA crosstalk. miRNAs have been reported as important regulators in plant and animal development, and some play essential roles in reproductive regulation [58,73]. This represents a new type of regulatory circuitry in which different types of RNAs can crosstalk with each other. In recent years, the functional target mimicries (or natural miRNA sponges) were initially discovered in plants [41], and subsequently in mammals, in which they were renamed to ceRNAs and were shown to be relevant in many process [55,56,74,75], suggesting that these molecules might represent a widespread form of gene regulation. Some lncRNAs that contain miRNA-binding sites have been shown to communicate with and regulate corresponding miRNA target genes by competing specifically for shared miRNAs. In plants, after the IPS1-target mimic of miR399 was identified, Wu *et al*. [42] predicted endogenous mimics (eTMs) for 20 conserved miRNAs from intergenic or non-coding gene-originated regions in *Arabidopsis* and rice, and several *Arabidopsis* eTMs have been shown to be functional [41,42,52]. We also predicted that lncRNAs act as ceRNAs for conserved miRNAs in rice. After experimental verification, two of these reproduction-related lncRNAs were confirmed to be target mimics of miR160 and miR164, respectively. It has been reported that a decrease in miR160 causes abnormal flower morphology, reduced fertility and aberrant seeds and that miR164 plays a role in specifying particular cell types during the later stages of flower development [61-63]. Considering that the ‘sponge’ of miR160 is highly expressed in early panicles and seeds after pollination and that the ‘sponge’ of miR164 is specifically expressed in pistil and anther, it is intriguing to associate these two lncRNAs with the functions of miR160/miR164 in regulating floral and/or seed development. We believe that the importance of lncRNAs in their role as ceRNAs during plant development and reproduction regulation will emerge within a few years.

## Conclusions

We identified 2,224 lncRNAs in rice, including both lincRNAs and lncNATs, with a focus on lncRNAs that are related to reproduction. The characteristics of rice lncRNAs were analyzed and compared with those of lncRNAs from other species. Further functional analysis showed that some lncRNAs function as ceRNAs and that one lncRNA functions as a regulator of panicle development and fertility. The research has provided a source of lncRNAs and associated insertional mutants in rice and has demonstrated the important functions played by lncRNAs in reproduction.

## Materials and methods

### Whole transcriptome library preparation and sequencing

Total RNA was obtained from rice anthers before flowering, pistils before flowering, spikelets 5 DAP and shoots 14 DAG; these samples were used for sequencing. The preparation of whole transcriptome libraries and deep sequencing were performed by the Annoroad Gene Technology Corporation (Beijing, PR China). Whole transcriptome libraries were constructed using TruSeq Stranded Total RNA with Ribo-Zero Gold (Illumina, San Diego, CA, USA) according to the manufacturer’s instructions. Libraries were controlled for quality and quantified using the BioAnalyzer 2100 system and qPCR (Kapa Biosystems, Woburn, MA, USA). The resulting libraries were sequenced initially on a HiSeq 2000 instrument that generated paired-end reads of 100 nucleotides. The sequencing data have been submitted to the NCBI Sequence Read Archive (SRA accession number SRP047482).

### Data sources

*Oryza sativa* genome assembly RGAP 7.0 was used throughout this study and was downloaded from [30]. All of the RNA-seq datasets used in this study were obtained from NCBI SRA. Detailed information on each RNA-seq dataset can be found in Additional file 1.

### lncRNA identification pipeline

The rice transcriptome was assembled using the Cufflinks 2.0 package according to the instructions provided [19]. Briefly, each RNA-seq dataset was aligned to the rice genome independently using the TopHat 2.0 program [76]. The transcriptome from each dataset was then assembled independently using the Cufflinks 2.0 program. All transcriptomes were pooled and merged to generate a final transcriptome using Cuffmerge. After the final transcriptome was produced, Cuffdiff was used to estimate the abundance of all transcripts based on the final transcriptome, and a BAM file was generated from the TopHat alignment. All transcripts without strand information and all single-exon transcripts within a range of 500 bp in the sense direction to other transcripts were discarded. Next, we discarded transcripts that overlapped with known mRNAs (including TE-related mRNAs) and transcripts with FPKM scores <0.5 (2 for single-exon transcripts) in all samples and transcripts shorter than 200 bp. Filtering of the remaining transcripts resulted in many novel, long, expressed transcripts. We first used the CPC [20] to predict transcripts with coding potential. All transcripts with CPC scores >0 were discarded. The remaining transcripts were subjected to HMMER [21] analysis to exclude transcripts that contained any known protein domains cataloged in the Pfam database [22]. The transcripts that remained were considered reliably expressed lncRNAs. The tissue-specific score (JS score) was calculated for each transcript using the csSpecificity() function in the CummeRbund R package [76].

### miRNA decoy site prediction

miRNA decoy sites were predicted using the algorithm developped by Wu *et al*. [42].

### Whole-genome alignment

A plant eight-way whole-genome alignment was performed according to the instructions of the UCSC Genome Browser Wiki [77]. Specifically, we first collected all of the necessary genome sequences, including those for *Musa acuminatea* (musa, v1.0) [31], *Arabidopsis thaliana* (*Arabidopsis*, TAIR9 assembly) [32], *Brachypodium distachyon* (brachypodium, v1.2) [33], *Zea mays* (maize, release 5b.60) [34], *Populus trichocarpa* (poplar, v2.2) [35], *Vitis vinifera* (grapevine, 12x) [36] and *Sorghum bicolor* (sorghum, v1.0) [37].

All eight genomes were masked for transposable elements and other simple repeats using the RepeatMasker program. As whole-genome alignment is computationally intensive, a Linux cluster was used for parallel computation. The phylogenic tree of the eight plants used for the multiple alignment was as follows: (((((Rice Brachypodium) Sorghum) Musa)) (Grapevine (Poplar *Arabidopsis*))). A phylogenic model was fitted based on the multiple alignment of the eight plant genomes using the phyloFit program [39] in the phastCons package [39]. The consScores of every base were calculated from the eight-way alignments based on the fitted model using the phastCons package.

### Insertion mutant analysis

To analyze the potential functions of the identified lincRNAs, we aligned the lincRNA sequences and their 1 kb upstream regions (for lincRNAs without strand information, both the downstream and upstream regions were used) to the FSTs of all of the mutants in nine rice mutant databases (Additional file 6) downloaded from RiceGE [78], which maintains these data on its ftp server. We retained mutants with sequence similarity scores >90% and a 5′ end located either in a lincRNA sequence or in the 1 kb upstream region of a lincRNA. The phenotypic information for the mutants from the Rice Tos17 Insertion Mutant Database [79] was obtained through a blast search of the original database using the identified FSTs, and phenotypic information linked to the blast results was collected manually.

### Gene ontology analysis

In accordance with previous studies, over-represented functional themes present in the genomic background were mapped onto the GO hierarchy using the Cytoscape plugin BINGO [80].

### Confirmation of lncRNA expression via qRT-PCR analysis

Total RNAs obtained from rice panicles both before and after heading, anthers before flowering, pistils before flowering, spikelets 5 DAP, embryos 25 DAP, calluses and the shoots and roots of 14-day-old seedlings were reverse transcribed using the PrimeScript™ RT reagent kit (Takara, Otsu, Shiga, Japan). Real-time PCR was performed using SYBR Premix Ex Taq™ (Takara) for amplification of the PCR products. Actin2 was chosen as a reference gene. Real-time PCR was conducted according to the manufacturer’s instructions (Takara), and the resultant melting curves were visually inspected to ensure the specificity of product detection. Quantification of lncRNA expression was performed using the comparative Ct method. These assays were performed in triplicate, and the results are presented as the mean ± standard deviations.

### *In situ* hybridization

*In situ* RNA hybridization was performed as described previously, with minor modifications [81]. Briefly, plant materials were fixed in FAA fixative for 8 h at 4°C, then dehydrated after vacuum infiltration using a graded ethanol series followed by a xylene series and embedded in Paraplast Plus (Sigma-Aldrich,St. Louis, MO, USA). Microtome sections (8 μm) were mounted on Probe-On™ Plus microscope slides (Fisher, Waltham, MA, USA), and lncRNAs were amplified, subcloned into the pEASY™-T3 (TransGen Biotech, Beijing, PR China) vector and used as templates to generate sense or antisense RNA probes. The probes were transcribed using T7/SP6 RNA polymerase. Digoxigenin-labeled RNA probes were prepared using the DIG RNA Labeling Kit (SP6/T7;Roche, Basel, Switzerland) according to the manufacturer’s instructions. Photomicrographs were obtained using a bright-field microscope (Leica DM5000B).

### Rice protoplast preparation, transfection, and RNA extraction

Protoplast isolation from rice green tissues was performed as previously described with some modifications [82,83]. Briefly, 14-day-old rice shoots were cut into approximately 0.5-mm strips and were incubated in enzyme solution (0.4 M sucrose, 20 mM KCL, 20 mM MES, 1% cellulase R-10 (Yakult Honsha,Tokyo, Japan), 0.4% macerozyme R-10 (Yakult Honsha), 10 mM CaCl_2_, 0.1% bovine serum albumin, 100 μg/ml Amp) for 4 to 5 h in the dark with gentle shaking (40 to 60 rpm). After digestion, the pellets were washed with W5 solution (154 mM NaCl, 125 mM CaCl_2_, 5 mM KCl and 2 mM MES, adjusted to pH 5.8 with KOH), and the protoplasts were collected by centrifugation at 1,500 g for 3 minutes. DNA (50 to 100 μg) was used to transfect every 1 ml (2 × 10^6^ cells) of rice protoplasts. Transfected protoplasts were incubated at 28°C for 24 h to allow RNA expression. Total RNA was then isolated from each sample using TRIzol (Invitrogen, Waltham, MA, USA) according to the manufacturer’s instructions.

## Additional files

Additional file 1: Table S1. RNA-seq datasets. Additional file 2: Table S2. Characteristics of all of the lncRNAs identified in this study. Additional file 3: Table S3. lncRNAs associated with small RNAs. Additional file 4: Figure S1. Properties of rice lncRNAs, related to Figure 2. **(A) **A/U content of the *Arabidopsis* lncRNA transcripts and various regions of protein-coding transcripts. **(B) **Distances of the lincRNAs, protein-coding genes and control regions from their closest protein-coding genes. The controls are random intergenic regions that were size and chromosome matched to the lincRNA set. **(C) **Lengths of the conserved segments in the exons of mRNAs and lincRNAs. **(D) **Relative orientations of the lincRNAs, protein-coding genes and control regions with respect to their closest protein-coding genes within 100,000 bases. The error bars indicate the standard deviation based on 1,000 cohorts of control regions, as described in (B). **(E) **Expression of LOC_Os08g35520.1 in wild-type plants and mutant plants. The total RNAs were extracted from the shoots 14 DAG and the young panicles before heading of the wild-type plants and the mutant plants, respectively, and then the expression of LOC_Os08g35520.1 was detected using RT-PCR. Actin2 was used as reference gene. Additional file 5: Figure S2. Enriched GO terms for protein-coding genes whose expression is correlated with reproductive and vegetative lincRNAs. Significantly overrepresented GO terms based on GO molecular functions and biological processes were visualized in Cytoscape [84]. The size of a node is proportional to the number of targets in the GO category. The color of the node represents the significance of enrichment: the deeper the color, the higher the enrichment significance. Reproduction-specific terms in the plot are highlighted. Additional file 6: Table S4. Detailed information on the rice insertional mutant databases used in this study. Additional file 7: Table S5. lncRNAs with available rice mutant resources. Additional file 8: Table S6. lncRNA mutants with apparent phenotypes. Additional file 9: Table S7. lncRNAs with miRNA decoy sites. Additional file 10:
**Putative miRNA binding sites in lncRNAs.**

## Abbreviations

AGO: Argonaute
ceRNA: competing endogenous RNA
CPC: Coding Potential Calculator
DAG: days after germination
DAP: days after pollination
FPKM: fragments per kilobase of transcript per million mapped reads
FST: flanking sequence tag
GO: gene ontology
JS: Jensen-Shannon
lincRNA: large intergenic non-coding RNA
lncNAT: long non-coding natural antisense transcript
lncRNA: long non-coding RNA
miRNA: microRNA
ncRNA: non-protein-coding RNA
PCR: polymerase chain reaction
qRT-PCR: quantitative RT-PCR
SRA: Sequence Read Archive
sRNA: small regulatory RNA
ssRNA-seq: strand-specific RNA sequencing
TE: transposable element
UTR: untranslated region
