# Supplementary figures and images for: Genome-wide screening and functional analysis identify a large number of long noncoding RNAs involved in the sexual reproduction of rice
Source: Genome Biol. 2014 Dec 3;15(12):512. doi: 10.1186/s13059-014-0512-1 (PMC4253996; doi:10.1186/s13059-014-0512-1)

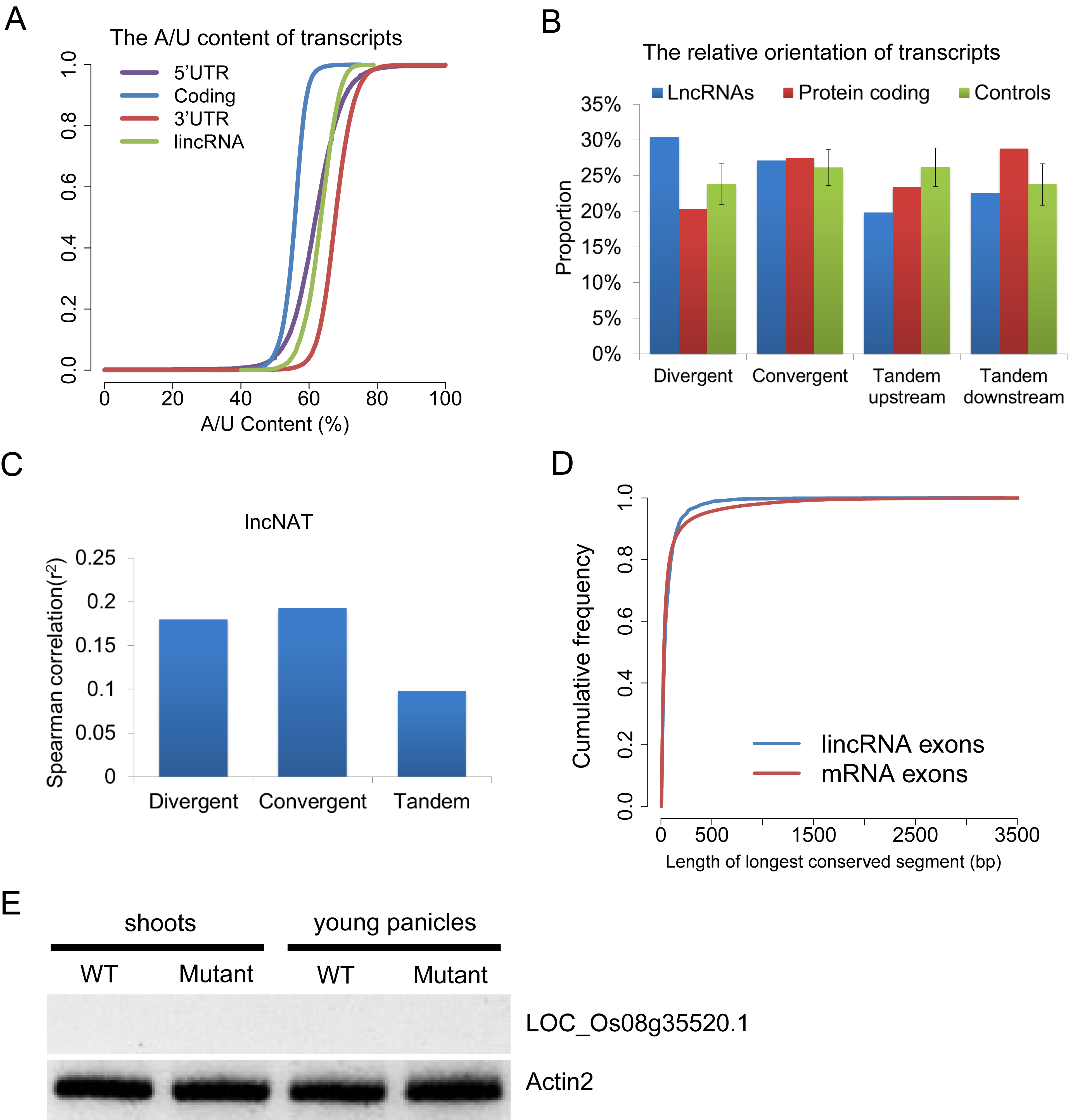

Supplement: Additional file 4: Figure S1. — Properties of rice lncRNAs, related to Figure 2. (A) A/U content of the Arabidopsis lncRNA transcripts and various regions of protein-coding transcripts. (B) Distances of the lincRNAs, protein-coding genes and control regions from their closest protein-coding genes. The controls are random intergenic regions that were size and chromosome matched to the lincRNA set. (C) Lengths of the conserved segments in the exons of mRNAs and lincRNAs. (D) Relative orientations of the lincRNAs, protein-coding genes and control regions with respect to their closest protein-coding genes within 100,000 bases. The error bars indicate the standard deviation based on 1,000 cohorts of control regions, as described in (B). (E) Expression of LOC_Os08g35520.1 in wild-type plants and mutant plants. The total RNAs were extracted from the shoots 14 DAG and the young panicles before heading of the wild-type plants and the mutant plants, respectively, and then the expression of LOC_Os08g35520.1 was detected using RT-PCR. Actin2 was used as reference gene. [file 13059_2014_512_MOESM4_ESM.jpeg]

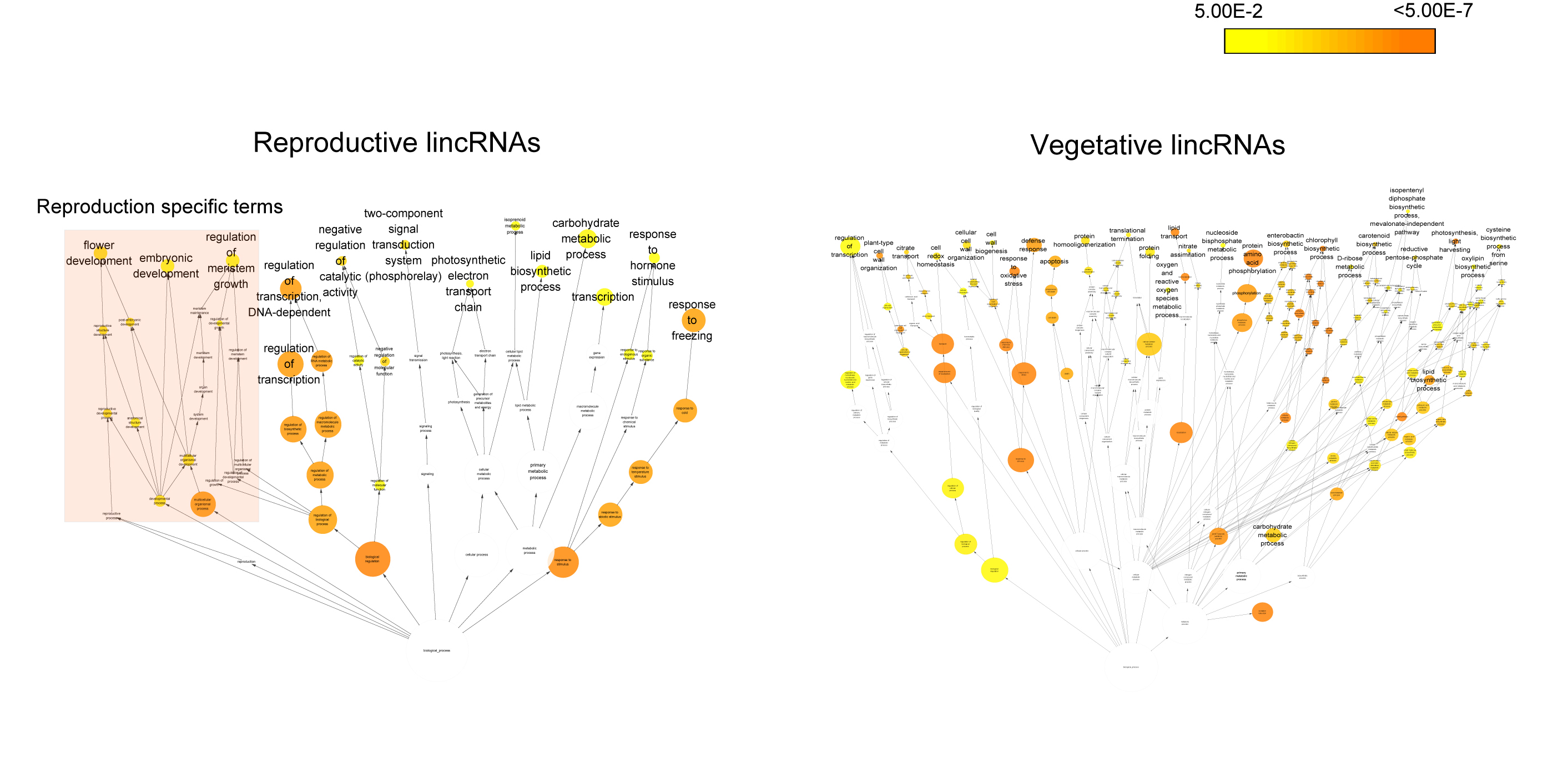

Supplement: Additional file 5: Figure S2. — Enriched GO terms for protein-coding genes whose expression is correlated with reproductive and vegetative lincRNAs. Significantly overrepresented GO terms based on GO molecular functions and biological processes were visualized in Cytoscape [84]. The size of a node is proportional to the number of targets in the GO category. The color of the node represents the significance of enrichment: the deeper the color, the higher the enrichment significance. Reproduction-specific terms in the plot are highlighted. [file 13059_2014_512_MOESM5_ESM.jpeg]
