# Supplementary material for: Genome-wide screening and functional analysis identify a large number of long noncoding RNAs involved in the sexual reproduction of rice
Source: Genome Biol. 2014 Dec 3;15(12):512. doi: 10.1186/s13059-014-0512-1 (PMC4253996; doi:10.1186/s13059-014-0512-1)
Supplement: Additional file 6: Table S4. — Detailed information on the rice insertional mutant databases used in this study. [file 13059_2014_512_MOESM6_ESM.docx]

|  | Database name | Link |
| --- | --- | --- |
| affjp | Rice Tos17 Insertion Mutant Database | <http://tos.nias.affrc.go.jp/index.html.en> |
| rmd | Rice Mutant Database | <http://rmd.ncpgr.cn/index.cgi> |
| ostid | - | <http://orygenesdb.cirad.fr/index.html> |
| ucd | Rice Transposon Flanking Sequence Tag Database | <http://sundarlab.ucdavis.edu/rice/blast/blast.html> |
| gsnu | - | <http://nongae.gsnu.ac.kr/~tbcg/src/pmbbrc.htm> |
| cirad | Oryza Tag Line | <http://oryzatagline.cirad.fr/> |
| ship | Shanghai T-DNA Insertion Population | <http://ship.plantsignal.cn/listGene.do> |
| pfg | Rice T-DNA Insertion Seqence Database | <http://www.postech.ac.kr/life/pfg/risd/> |
| trim | Taiwan Rice Insertional Mutants Database | <http://trim.sinica.edu.tw/> |
