# Supplementary material for: Genome-wide screening and functional analysis identify a large number of long noncoding RNAs involved in the sexual reproduction of rice
Source: Genome Biol. 2014 Dec 3;15(12):512. doi: 10.1186/s13059-014-0512-1 (PMC4253996; doi:10.1186/s13059-014-0512-1)
Supplement: Additional file 10: — Putative miRNA binding sites in lncRNAs. [file 13059_2014_512_MOESM10_ESM.pdf]

```
>osa-miR395h MIMAT0000963   Score: 4.5  TCONS_00100257 gene=XLOC_057032

Query:          1 GTGAAGTGT--T-TGGGGGAACTC 21
                  |||||:|:|**|*|||:|:||||*
Sbjct:       1232 CACTTTATAGTACACCTCTTTGAT 1209


>osa-miR156b-5p MIMAT0000619    Score: 2.0  TCONS_00043170 gene=XLOC_024537

Query:          1 TGACAGAAG--A-GAGTGAGCAC 20
                  ||||||||:**|*||||||||||
Sbjct:        719 ACTGTCTTT CTCCTCACTCGTG 697


>osa-miR156b-5p MIMAT0000619    Score: 2.0  TCONS_00043171 gene=XLOC_024537

Query:          1 TGACAGAAG--A-GAGTGAGCAC 20
                  ||||||||:**|*||||||||||
Sbjct:        719 ACTGTCTTT CTCCTCACTCGTG 697


>osa-miR156b-5p MIMAT0000619    Score: 2.0  TCONS_00043172 gene=XLOC_024537

Query:          1 TGACAGAAG--A-GAGTGAGCAC 20
                  ||||||||:**|*||||||||||
Sbjct:        719 ACTGTCTTT CTCCTCACTCGTG 697


>osa-miR156b-5p MIMAT0000619    Score: 2.0  TCONS_00043174 gene=XLOC_024537

Query:          1 TGACAGAAG--A-GAGTGAGCAC 20
                  ||||||||:**|*||||||||||
Sbjct:        719 ACTGTCTTT CTCCTCACTCGTG 697


>osa-miR156b-5p MIMAT0000619    Score: 2.0  TCONS_00043175 gene=XLOC_024537

Query:          1 TGACAGAAG--A-GAGTGAGCAC 20
                  ||||||||:**|*||||||||||
Sbjct:        719 ACTGTCTTT CTCCTCACTCGTG 697


>osa-miR156b-5p MIMAT0000619    Score: 2.0  TCONS_00043176 gene=XLOC_024537
```

```
Query:           1 TGACAGAAG--A-GAGTGAGCAC 20
                   ||||||||:**|*||||||||||
Sbjct:         719 ACTGTCTTT CTCCTCACTCGTG 697
```

```
>osa-miR156b-5p MIMAT0000619    Score: 4.5  TCONS_00076760 gene=XLOC_043053

Query:           1 TGACAGAAG-AG--AGTGAGCAC 20
                   ||*||||||*||**||||||*|*
Sbjct:         398 ACCGTCTTCCTCTTTCACTCCTT 376
```

```
>osa-miR156b-5p MIMAT0000619    Score: 4.5  TCONS_00087104 gene=XLOC_049223

Query:           1 TGACAGAAG-AG--AGTGAGCAC 20
                   ||||||||**||**|||:|:||*
Sbjct:        1646 ACTGTCTTACTCT TCATTTGTT 1624
```

```
>osa-miR395p MIMAT0003873   Score: 4.5  TCONS_00100257 gene=XLOC_057032

Query:           1 GTGAAGTGT--T-TGGGGGAACTC 21
                   |||||:|:|**|*|||:|:||||*
Sbjct:        1232 CACTTTATAGTACACCTCTTTGAT 1209
```

```
>osa-miR395y MIMAT0014125   Score: 4.5  TCONS_00100257 gene=XLOC_057032

Query:           1 GTGAAGTGT--T-TGGGGGAACTC 21
                   |||||:|:|**|*|||:|:||||*
Sbjct:        1232 CACTTTATAGTACACCTCTTTGAT 1209
```

```
>osa-miR169r-5p MIMAT0026762    Score: 4.0  TCONS_00086804 gene=XLOC_049024

Query:           1 TAGCCAAGG-AT--GATTTGCCTG 21
                   ||:|||||:*||**|||||||*:|
Sbjct:        5288 ATTGGTTCTATATCCTAAACGAGC 5265
```

```
>osa-miR169r-5p MIMAT0026762    Score: 4.5  TCONS_00087224 gene=XLOC_049308

Query:           1 TAGCCAAGG-AT--GATTTGCCTG 21
                   :||||||*|*||**||||*||||:
```

```
Sbjct:        690 GTCGGTTACATATCCTAACCGGAT 667


>osa-miR169r-5p MIMAT0026762    Score: 4.5  TCONS_00087226 gene=XLOC_049308

Query:          1 TAGCCAAGG-AT--GATTTGCCTG 21
                  :||||||*|*||**||||*||||:
Sbjct:        690 GTCGGTTACATATCCTAACCGGAT 667


>osa-miR169r-5p MIMAT0026762    Score: 4.5  TCONS_00087227 gene=XLOC_049308

Query:          1 TAGCCAAGG-AT--GATTTGCCTG 21
                  :||||||*|*||**||||*||||:
Sbjct:        704 GTCGGTTACATATCCTAACCGGAT 681


>osa-miR156h-3p MIMAT0031157    Score: 4.5  TCONS_00098451 gene=XLOC_055866

Query:          1 GCTCACTTCT--C-TTTCTGTCAGC 22
                  ||*|||||||**|*|||||||||**
Sbjct:        758 CGTGTGAAGAGTGTAAAGACAGTGA 734


>osa-miR156g-5p MIMAT0000624    Score: 2.0  TCONS_00043170 gene=XLOC_024537

Query:          1 TGACAGAAG--A-GAGTGAGCAC 20
                  ||||||||:**|*||||||||||
Sbjct:        719 ACTGTCTTT CTCCTCACTCGTG 697


>osa-miR156g-5p MIMAT0000624    Score: 2.0  TCONS_00043171 gene=XLOC_024537

Query:          1 TGACAGAAG--A-GAGTGAGCAC 20
                  ||||||||:**|*||||||||||
Sbjct:        719 ACTGTCTTT CTCCTCACTCGTG 697


>osa-miR156g-5p MIMAT0000624    Score: 2.0  TCONS_00043172 gene=XLOC_024537

Query:          1 TGACAGAAG--A-GAGTGAGCAC 20
                  ||||||||:**|*||||||||||
Sbjct:        719 ACTGTCTTT CTCCTCACTCGTG 697
```

```
>osa-miR156g-5p MIMAT0000624    Score: 2.0  TCONS_00043174 gene=XLOC_024537

Query:           1 TGACAGAAG--A-GAGTGAGCAC 20
                     ||||||||:**|*|||||||||
Sbjct:         719 ACTGTCTTT CTCCTCACTCGTG 697


>osa-miR156g-5p MIMAT0000624    Score: 2.0  TCONS_00043175 gene=XLOC_024537

Query:           1 TGACAGAAG--A-GAGTGAGCAC 20
                     ||||||||:**|*|||||||||
Sbjct:         719 ACTGTCTTT CTCCTCACTCGTG 697


>osa-miR156g-5p MIMAT0000624    Score: 2.0  TCONS_00043176 gene=XLOC_024537

Query:           1 TGACAGAAG--A-GAGTGAGCAC 20
                     ||||||||:**|*|||||||||
Sbjct:         719 ACTGTCTTT CTCCTCACTCGTG 697


>osa-miR156g-5p MIMAT0000624    Score: 4.5  TCONS_00076760 gene=XLOC_043053

Query:           1 TGACAGAAG-AG--AGTGAGCAC 20
                     ||*||||||*||**||||||*|*
Sbjct:         398 ACCGTCTTCCTCTTTCACTCCTT 376


>osa-miR156g-5p MIMAT0000624    Score: 4.5  TCONS_00087104 gene=XLOC_049223

Query:           1 TGACAGAAG-AG--AGTGAGCAC 20
                     ||||||||**||**|||:|:||*
Sbjct:        1646 ACTGTCTTACTCT TCATTTGTT 1624


>osa-miR159d MIMAT0001025   Score: 4.5  TCONS_00043583 gene=XLOC_024724

Query:           1 ATTGGATTG-A--AGGGAGCTCCG 21
                     |:|||||||*|**||:||*||*||
Sbjct:         700 TGACCTAACCTCCTCTCTGGAAGC 677


>osa-miR159d MIMAT0001025   Score: 4.5  TCONS_00043584 gene=XLOC_024724
```

```
Query:           1 ATTGGATTG-A--AGGGAGCTCCG 21
                   |:|||||||*|**||:||*||*||
Sbjct:         700 TGACCTAACCTCCTCTCTGGAAGC 677
```

>osa-miR159d MIMAT0001025   Score: 4.5 TCONS_00043585 gene=XLOC_024724

```
Query:           1 ATTGGATTG-A--AGGGAGCTCCG 21
                   |:|||||||*|**||:||*||*||
Sbjct:         700 TGACCTAACCTCCTCTCTGGAAGC 677
```

>osa-miR159d MIMAT0001025   Score: 4.5 TCONS_00043586 gene=XLOC_024724

```
Query:           1 ATTGGATTG-A--AGGGAGCTCCG 21
                   |:|||||||*|**||:||*||*||
Sbjct:         827 TGACCTAACCTCCTCTCTGGAAGC 804
```

>osa-miR160a-5p MIMAT0000628   Score: 3.5 TCONS_00111350 gene=XLOC_063639

```
Query:           1 TGCCTGGCT---CCCTGTATGCCA 21
                   |||||||||***||*||||||||*
Sbjct:         532 ACGGACCGAAAGGGAACATACGGC 509
```

>osa-miR166c-5p MIMAT0022857   Score: 4.5 TCONS_00072868 gene=XLOC_040881

```
Query:           1 GGAATGTTG-T--CTGGTCCGAG 20
                   |:|||||||*|**|||||*|*|:
Sbjct:        2966 CTTTACAACAATCGACCACGGTT 2944
```

>osa-miR166c-5p MIMAT0022857   Score: 4.5 TCONS_00072869 gene=XLOC_040881

```
Query:           1 GGAATGTTG-T--CTGGTCCGAG 20
                   |:|||||||*|**|||||*|*|:
Sbjct:        3719 CTTTACAACAATCGACCACGGTT 3697
```

>osa-miR166c-5p MIMAT0022857   Score: 4.5 TCONS_00102306 gene=XLOC_058088

```
Query:           1 GGAATGTTGT--C-TGGTCCGAG 20
```

```
                  |||||||::|**|*||||*|:|:
Sbjct:        452 CCTTACAGTACTGTACCACGTTT 430


>osa-miR156l-5p MIMAT0001021    Score: 4.5  TCONS_00043170 gene=XLOC_024537

Query:          1 CGACAGAAG--A-GAGTGAGCATA 21
                  *|||||||:**|*|||||||||:*
Sbjct:        719 ACTGTCTTT CTCCTCACTCGTGC 696


>osa-miR156l-5p MIMAT0001021    Score: 4.5  TCONS_00043171 gene=XLOC_024537

Query:          1 CGACAGAAG--A-GAGTGAGCATA 21
                  *|||||||:**|*|||||||||:*
Sbjct:        719 ACTGTCTTT CTCCTCACTCGTGC 696


>osa-miR156l-5p MIMAT0001021    Score: 4.5  TCONS_00043172 gene=XLOC_024537

Query:          1 CGACAGAAG--A-GAGTGAGCATA 21
                  *|||||||:**|*|||||||||:*
Sbjct:        719 ACTGTCTTT CTCCTCACTCGTGC 696


>osa-miR156l-5p MIMAT0001021    Score: 4.5  TCONS_00043174 gene=XLOC_024537

Query:          1 CGACAGAAG--A-GAGTGAGCATA 21
                  *|||||||:**|*|||||||||:*
Sbjct:        719 ACTGTCTTT CTCCTCACTCGTGC 696


>osa-miR156l-5p MIMAT0001021    Score: 4.5  TCONS_00043175 gene=XLOC_024537

Query:          1 CGACAGAAG--A-GAGTGAGCATA 21
                  *|||||||:**|*|||||||||:*
Sbjct:        719 ACTGTCTTT CTCCTCACTCGTGC 696


>osa-miR156l-5p MIMAT0001021    Score: 4.5  TCONS_00043176 gene=XLOC_024537

Query:          1 CGACAGAAG--A-GAGTGAGCATA 21
                  *|||||||:**|*|||||||||:*
Sbjct:        719 ACTGTCTTT CTCCTCACTCGTGC 696
```

```
>osa-miR160f-3p MIMAT0022868    Score: 4.5  TCONS_00009919 gene=XLOC_005247

Query:          1 GCATTGAGGG-A--GTCATGCAGG 21
                    ||||||||||*|**:|||*|||*:
Sbjct:        596 CGTAACTCCCTTCGTAGTTCGTAT 573


>osa-miR166i-3p MIMAT0001074    Score: 4.0  TCONS_00068571 gene=XLOC_038526

Query:          1 TCGGATCAG---GCTTCATTCCTC 21
                    |||||:||:***||||*|||||:|
Sbjct:        839 AGCCTGGTTAAGCGAACTAAGGGG 816


>osa-miR160b-5p MIMAT0000629    Score: 3.5  TCONS_00111350 gene=XLOC_063639

Query:          1 TGCCTGGCT---CCCTGTATGCCA 21
                    |||||||||***||*||||||||*
Sbjct:        532 ACGGACCGAAAGGGAACATACGGC 509


>osa-miR166b-3p MIMAT0000636    Score: 3.0  TCONS_00068571 gene=XLOC_038526

Query:          1 TCGGACCAG---GCTTCATTCCCC 21
                    ||||||||:***||||*|||||||
Sbjct:        839 AGCCTGGTTAAGCGAACTAAGGGG 816


>osa-miR164b MIMAT0000634   Score: 4.5  TCONS_00100980 gene=XLOC_057427

Query:          1 TGGAGAAGC-A-G-GGCACGTGCA 21
                    ||||:||||*|*|*:||||::|*|
Sbjct:        732 ACCTTTTCGTTGCGTCGTGTGCT 709


>osa-miR166a-3p MIMAT0000635    Score: 3.0  TCONS_00068571 gene=XLOC_038526

Query:          1 TCGGACCAG---GCTTCATTCCCC 21
                    ||||||||:***||||*|||||||
Sbjct:        839 AGCCTGGTTAAGCGAACTAAGGGG 816
```

```
>osa-miR166g-3p MIMAT0001072    Score: 3.5  TCONS_00068571 gene=XLOC_038526

Query:           1 TCGGACCAG---GCTTCATTCCTC 21
                     ||||||||:***||||*|||||:|
Sbjct:         839 AGCCTGGTTAAGCGAACTAAGGGG 816


>osa-miR156f-3p MIMAT0022847    Score: 4.5  TCONS_00098451 gene=XLOC_055866

Query:           1 GCTCACTTCT--C-TTTCTGTCAGC 22
                     ||*|||||||**|*|||||||||**
Sbjct:         758 CGTGTGAAGAGTGTAAAGACAGTGA 734


>osa-miR395q MIMAT0003874   Score: 4.5  TCONS_00100257 gene=XLOC_057032

Query:           1 GTGAAGTGT--T-TGGGGGAACTC 21
                     |||||:|:|**|*|||:|:||||*
Sbjct:        1232 CACTTTATAGTACACCTCTTTGAT 1209


>osa-miR167d-3p MIMAT0022872    Score: 4.5  TCONS_00026523 gene=XLOC_014915

Query:           1 GATCATGCT--G-TGCAGTTTCATC 22
                     *||||||||**|*|*||||||||*|
Sbjct:         901 GTAGTACGAGACAAGGTCAAAGTTG 877


>osa-miR156j-5p MIMAT0000627    Score: 2.0  TCONS_00043170 gene=XLOC_024537

Query:           1 TGACAGAAG--A-GAGTGAGCAC 20
                     ||||||||:**|*||||||||||
Sbjct:         719 ACTGTCTTT CTCCTCACTCGTG 697


>osa-miR156j-5p MIMAT0000627    Score: 2.0  TCONS_00043171 gene=XLOC_024537

Query:           1 TGACAGAAG--A-GAGTGAGCAC 20
                     ||||||||:**|*||||||||||
Sbjct:         719 ACTGTCTTT CTCCTCACTCGTG 697


>osa-miR156j-5p MIMAT0000627    Score: 2.0  TCONS_00043172 gene=XLOC_024537
```

Query: 1 TGACAGAAG--A-GAGTGAGCAC 20
||||||||:**|*||||||||||
Sbjct: 719 ACTGTCTTT CTCCTCACTCGTG 697

>osa-miR156j-5p MIMAT0000627 Score: 2.0 TCONS_00043174 gene=XLOC_024537

Query: 1 TGACAGAAG--A-GAGTGAGCAC 20
||||||||:**|*||||||||||
Sbjct: 719 ACTGTCTTT CTCCTCACTCGTG 697

>osa-miR156j-5p MIMAT0000627 Score: 2.0 TCONS_00043175 gene=XLOC_024537

Query: 1 TGACAGAAG--A-GAGTGAGCAC 20
||||||||:**|*||||||||||
Sbjct: 719 ACTGTCTTT CTCCTCACTCGTG 697

>osa-miR156j-5p MIMAT0000627 Score: 2.0 TCONS_00043176 gene=XLOC_024537

Query: 1 TGACAGAAG--A-GAGTGAGCAC 20
||||||||:**|*||||||||||
Sbjct: 719 ACTGTCTTT CTCCTCACTCGTG 697

>osa-miR156j-5p MIMAT0000627 Score: 4.5 TCONS_00076760 gene=XLOC_043053

Query: 1 TGACAGAAG-AG--AGTGAGCAC 20
||*||||||*||**||||||*|*
Sbjct: 398 ACCGTCTTCCTCTTTCACTCCTT 376

>osa-miR156j-5p MIMAT0000627 Score: 4.5 TCONS_00087104 gene=XLOC_049223

Query: 1 TGACAGAAG-AG--AGTGAGCAC 20
||||||||**||**|||:|:||*
Sbjct: 1646 ACTGTCTTACTCT TCATTTGTT 1624

>osa-miR395e MIMAT0000961 Score: 4.5 TCONS_00100257 gene=XLOC_057032

Query: 1 GTGAAGTGT--T-TGGGGGAACTC 21
|||||:|:|**|*|||:|:||||*

```
Sbjct:       1232 CACTTTATAGTACACCTCTTTGAT 1209


>osa-miR160c-5p MIMAT0000630    Score: 3.5  TCONS_00111350 gene=XLOC_063639

Query:          1 TGCCTGGCT---CCCTGTATGCCA 21
                  |||||||||***||*||||||||*
Sbjct:        532 ACGGACCGAAAGGGAACATACGGC 509


>osa-miR395m MIMAT0003870   Score: 4.5  TCONS_00100257 gene=XLOC_057032

Query:          1 GTGAAGTGT--T-TGGGGGAACTC 21
                  |||||:|:|**|*|||:|:||||*
Sbjct:       1232 CACTTTATAGTACACCTCTTTGAT 1209


>osa-miR319a-5p MIMAT0020914    Score: 4.5  TCONS_00022704 gene=XLOC_012605

Query:          1 AGCTGCCGAA-T--CATCCATTCA 21
                  |||:|||:*|*|**|||||||||*
Sbjct:       2333 TCGGCGGTGTGATGGTAGGTAAGA 2310


>osa-miR166k-3p MIMAT0001037    Score: 4.5  TCONS_00011308 gene=XLOC_006054

Query:          1 TCGGACCAG---GCTTCAATCCCT 21
                  ||||||||****||:|||||*||:
Sbjct:        462 AGCCTGGTACTGCGGAGTTAAGGG 439


>osa-miR166k-3p MIMAT0001037    Score: 4.5  TCONS_00068571 gene=XLOC_038526

Query:          1 TCGGACCAG---GCTTCAATCCCT 21
                  ||||||||:***||||*|*||||:
Sbjct:        839 AGCCTGGTTAAGCGAACTAAGGGG 816


>osa-miR1561-3p MIMAT0022866    Score: 4.5  TCONS_00098451 gene=XLOC_055866

Query:          1 GCTCACTTCT--C-TTTCTGTCAGC 22
                  ||*|||||||**|*|||||||||**
Sbjct:        758 CGTGTGAAGAGTGTAAAGACAGTGA 734
```

```
>osa-miR166d-3p MIMAT0000638    Score: 3.0  TCONS_00068571 gene=XLOC_038526

Query:          1 TCGGACCAG---GCTTCATTCCCC 21
                  ||||||||:***||||*|||||||
Sbjct:        839 AGCCTGGTTAAGCGAACTAAGGGG 816


>osa-miR156e MIMAT0000622   Score: 2.0  TCONS_00043170 gene=XLOC_024537

Query:          1 TGACAGAAG--A-GAGTGAGCAC 20
                  ||||||||:**|*||||||||||
Sbjct:        719 ACTGTCTTT CTCCTCACTCGTG 697


>osa-miR156e MIMAT0000622   Score: 2.0  TCONS_00043171 gene=XLOC_024537

Query:          1 TGACAGAAG--A-GAGTGAGCAC 20
                  ||||||||:**|*||||||||||
Sbjct:        719 ACTGTCTTT CTCCTCACTCGTG 697


>osa-miR156e MIMAT0000622   Score: 2.0  TCONS_00043172 gene=XLOC_024537

Query:          1 TGACAGAAG--A-GAGTGAGCAC 20
                  ||||||||:**|*||||||||||
Sbjct:        719 ACTGTCTTT CTCCTCACTCGTG 697


>osa-miR156e MIMAT0000622   Score: 2.0  TCONS_00043174 gene=XLOC_024537

Query:          1 TGACAGAAG--A-GAGTGAGCAC 20
                  ||||||||:**|*||||||||||
Sbjct:        719 ACTGTCTTT CTCCTCACTCGTG 697


>osa-miR156e MIMAT0000622   Score: 2.0  TCONS_00043175 gene=XLOC_024537

Query:          1 TGACAGAAG--A-GAGTGAGCAC 20
                  ||||||||:**|*||||||||||
Sbjct:        719 ACTGTCTTT CTCCTCACTCGTG 697


>osa-miR156e MIMAT0000622   Score: 2.0  TCONS_00043176 gene=XLOC_024537
```

```
Query:          1 TGACAGAAG--A-GAGTGAGCAC 20
                  ||||||||:**|*||||||||||
Sbjct:        719 ACTGTCTTT CTCCTCACTCGTG 697


>osa-miR156e MIMAT0000622   Score: 4.5  TCONS_00076760 gene=XLOC_043053

Query:          1 TGACAGAAG-AG--AGTGAGCAC 20
                  ||*||||||*||**||||||*|*
Sbjct:        398 ACCGTCTTCCTCTTTCACTCCTT 376


>osa-miR156e MIMAT0000622   Score: 4.5  TCONS_00087104 gene=XLOC_049223

Query:          1 TGACAGAAG-AG--AGTGAGCAC 20
                  ||||||||**||**|||:|:||*
Sbjct:       1646 ACTGTCTTACTCT TCATTTGTT 1624


>osa-miR156d MIMAT0000621   Score: 2.0  TCONS_00043170 gene=XLOC_024537

Query:          1 TGACAGAAG--A-GAGTGAGCAC 20
                  ||||||||:**|*||||||||||
Sbjct:        719 ACTGTCTTT CTCCTCACTCGTG 697


>osa-miR156d MIMAT0000621   Score: 2.0  TCONS_00043171 gene=XLOC_024537

Query:          1 TGACAGAAG--A-GAGTGAGCAC 20
                  ||||||||:**|*||||||||||
Sbjct:        719 ACTGTCTTT CTCCTCACTCGTG 697


>osa-miR156d MIMAT0000621   Score: 2.0  TCONS_00043172 gene=XLOC_024537

Query:          1 TGACAGAAG--A-GAGTGAGCAC 20
                  ||||||||:**|*||||||||||
Sbjct:        719 ACTGTCTTT CTCCTCACTCGTG 697


>osa-miR156d MIMAT0000621   Score: 2.0  TCONS_00043174 gene=XLOC_024537

Query:          1 TGACAGAAG--A-GAGTGAGCAC 20
```

||||||||:**|*||||||||||
Sbjct: 719 ACTGTCTTT CTCCTCACTCGTG 697

>osa-miR156d MIMAT0000621 Score: 2.0 TCONS_00043175 gene=XLOC_024537

Query: 1 TGACAGAAG--A-GAGTGAGCAC 20
||||||||:**|*||||||||||
Sbjct: 719 ACTGTCTTT CTCCTCACTCGTG 697

>osa-miR156d MIMAT0000621 Score: 2.0 TCONS_00043176 gene=XLOC_024537

Query: 1 TGACAGAAG--A-GAGTGAGCAC 20
||||||||:**|*||||||||||
Sbjct: 719 ACTGTCTTT CTCCTCACTCGTG 697

>osa-miR156d MIMAT0000621 Score: 4.5 TCONS_00076760 gene=XLOC_043053

Query: 1 TGACAGAAG-AG--AGTGAGCAC 20
||*||||||*||**||||||*|*
Sbjct: 398 ACCGTCTTCCTCTTTCACTCCTT 376

>osa-miR156d MIMAT0000621 Score: 4.5 TCONS_00087104 gene=XLOC_049223

Query: 1 TGACAGAAG-AG--AGTGAGCAC 20
||||||||**||**|||:|:||*
Sbjct: 1646 ACTGTCTTACTCT TCATTTGTT 1624

>osa-miR529a MIMAT0002885 Score: 4.5 TCONS_00007478 gene=XLOC_003882

Query: 1 CTGTACCCT-CT--CTCTTCTTC 20
|||||**|:*||**||||||:||
Sbjct: 738 GACATTCGGTGAGTGAGAAGGAG 716

>osa-miR529a MIMAT0002885 Score: 4.5 TCONS_00030527 gene=XLOC_017512

Query: 1 CTGTACCCT---CTCTCTTCTTC 20
|||*|*||****|||||||||||
Sbjct: 249 GACTTTGGTGGTGAGAGAAGAAG 227

```
>osa-miR529a MIMAT0002885   Score: 4.5  TCONS_00039632 gene=XLOC_022908

Query:           1 CTGTACCCT-C-T-CTCTTCTTC 20
                   |:|:*||*|*|*|*|||||||||
Sbjct:         668 GGCGCGGAAGGAAGGAGAAGAAG 646


>osa-miR529a MIMAT0002885   Score: 3.5  TCONS_00056638 gene=XLOC_031432

Query:           1 CTGTACCCT-C-T-CTCTTCTTC 20
                   ||:||*|||*|*|*|||:|||||
Sbjct:         337 GATATTGGAAGAAGGAGGAGAAG 315


>osa-miR529a MIMAT0002885   Score: 4.5  TCONS_00056928 gene=XLOC_031563

Query:           1 CTGTACCCT-CT--CTCTTCTTC 20
                   ||*|*||||*||**||||:|:||
Sbjct:          27 GAGAGGGGAAGAGGGAGAGGGAG 5


>osa-miR408-5p MIMAT0022884 Score: 4.5  TCONS_00086475 gene=XLOC_048799

Query:           1 CAGGGATGA-G--GCAGAGCATGG 21
                   *||||||||*|**|||||*|||*|
Sbjct:         790 ATCCCTACT CGACGTCTAGTAAC 767


>osa-miR2118c MIMAT0011742  Score: 4.5  TCONS_00044208 gene=XLOC_025107

Query:           1 TTCCCGATG-CC--TCCTATTCCTA 22
                   |||*|:||**||**|||||||||:|
Sbjct:        1124 AAGTGTTAGAGGATAGGATAAGGGT 1100


>osa-miR164a MIMAT0000633   Score: 4.5  TCONS_00100980 gene=XLOC_057427

Query:           1 TGGAGAAGC-A-G-GGCACGTGCA 21
                   ||||:||||*|*|*:||||::|*|
Sbjct:         732 ACCTTTTCGTTGCGTCGTGTGC T 709
```

```
>osa-miR166m MIMAT0001087   Score: 3.5  TCONS_00068571 gene=XLOC_038526

Query:           1 TCGGACCAG---GCTTCATTCCCT 21
                     ||||||||:***||||*||||||:
Sbjct:         839 AGCCTGGTTAAGCGAACTAAGGGG 816


>osa-miR528-5p MIMAT0002884 Score: 4.5  TCONS_00010253 gene=XLOC_005464

Query:           1 TGGAAGGGGC-A--TGCAGAGGAG 21
                     :||*|||:||*|**|||*||||||
Sbjct:         256 GCCGTCCTCGTTGAACGACTCCTC 233


>osa-miR528-5p MIMAT0002884 Score: 4.5  TCONS_00010254 gene=XLOC_005464

Query:           1 TGGAAGGGGC-A--TGCAGAGGAG 21
                     :||*|||:||*|**|||*||||||
Sbjct:         542 GCCGTCCTCGTTGAACGACTCCTC 519


>osa-miR528-5p MIMAT0002884 Score: 4.5  TCONS_00010255 gene=XLOC_005464

Query:           1 TGGAAGGGGC-A--TGCAGAGGAG 21
                     :||*|||:||*|**|||*||||||
Sbjct:         542 GCCGTCCTCGTTGAACGACTCCTC 519


>osa-miR528-5p MIMAT0002884 Score: 4.5  TCONS_00004039 gene=XLOC_002180

Query:           1 TGGAAGGGGC--A-TGCAGAGGAG 21
                     ||||*|||||**|*||*||*||||
Sbjct:         354 ACCTCCCCCGCTTGACCTCGCCTC 331


>osa-miR528-5p MIMAT0002884 Score: 4.5  TCONS_00045558 gene=XLOC_025866

Query:           1 TGGAAGGGG---CATGCAGAGGAG 21
                     |*:||||:|***||||||||*|||
Sbjct:        1453 AATTTCCTCAGAGTACGTCTACTC 1430


>osa-miR528-5p MIMAT0002884 Score: 4.5  TCONS_00054114 gene=XLOC_030298
```

```
Query:          1 TGGAAGGGGC-A--TGCAGAGGAG 21
                  :||*|||:||*|**|||*||||||
Sbjct:        484 GCCGTCCTCGCTGAACGACTCCTC 461


>osa-miR528-5p MIMAT0002884 Score: 4.5  TCONS_00054115 gene=XLOC_030298

Query:          1 TGGAAGGGGC-A--TGCAGAGGAG 21
                  :||*|||:||*|**|||*||||||
Sbjct:        484 GCCGTCCTCGCTGAACGACTCCTC 461


>osa-miR528-5p MIMAT0002884 Score: 4.5  TCONS_00054116 gene=XLOC_030298

Query:          1 TGGAAGGGGC-A--TGCAGAGGAG 21
                  :||*|||:||*|**|||*||||||
Sbjct:        484 GCCGTCCTCGCTGAACGACTCCTC 461


>osa-miR528-5p MIMAT0002884 Score: 4.5  TCONS_00054117 gene=XLOC_030298

Query:          1 TGGAAGGGGC-A--TGCAGAGGAG 21
                  :||*|||:||*|**|||*||||||
Sbjct:        484 GCCGTCCTCGCTGAACGACTCCTC 461


>osa-miR395o MIMAT0003872   Score: 4.5  TCONS_00045975 gene=XLOC_026103

Query:          1 ATGAAGTGT-TT--GGAGGAACTC 21
                  ||||*||||*||***:||||||:|
Sbjct:       1099 TACTACACATAATGGTTCCTTGGG 1076


>osa-miR395g MIMAT0000962   Score: 4.5  TCONS_00100257 gene=XLOC_057032

Query:          1 GTGAAGTGT--T-TGGGGGAACTC 21
                  |||||:|:|**|*|||:|:||||*
Sbjct:       1232 CACTTTATAGTACACCTCTTTGAT 1209


>osa-miR164d MIMAT0001034   Score: 4.5  TCONS_00013342 gene=XLOC_007028

Query:          1 TGGAGAAGC--A-GGGCACGTGCT 21
                  |:||:||||**|*|:||||:|*||
```

```
Sbjct:       1194 ATCTTTTCGTATGCTCGTGTAGGA 1171


>osa-miR395r MIMAT0003878   Score: 4.5  TCONS_00100257 gene=XLOC_057032

Query:          1 GTGAAGTGT--T-TGGGGGAACTC 21
                  |||||:|:|**|*|||:|:||||*
Sbjct:       1232 CACTTTATAGTACACCTCTTTGAT 1209


>osa-miR399k MIMAT0000994   Score: 4.5  TCONS_00038693 gene=XLOC_022332

Query:          1 TGCCAAAGG--A-AATTTGCCCCG 21
                  |||||||::**|*|||||*|||*
Sbjct:       1545 ACGGTTTTTTTATTAAACAGGGG 1522


>osa-miR399k MIMAT0000994   Score: 4.5  TCONS_00038694 gene=XLOC_022332

Query:          1 TGCCAAAGG--A-AATTTGCCCCG 21
                  |||||||::**|*|||||*|||*
Sbjct:       1943 ACGGTTTTTTTATTAAACAGGGG 1920


>osa-miR399k MIMAT0000994   Score: 4.5  TCONS_00038695 gene=XLOC_022332

Query:          1 TGCCAAAGG--A-AATTTGCCCCG 21
                  |||||||::**|*|||||*|||*
Sbjct:       1940 ACGGTTTTTTTATTAAACAGGG  1917


>osa-miR395i MIMAT0000964   Score: 4.5  TCONS_00100257 gene=XLOC_057032

Query:          1 GTGAAGTGT--T-TGGGGGAACTC 21
                  |||||:|:|**|*|||:|:||||*
Sbjct:       1232 CACTTTATAGTACACCTCTTTGAT 1209


>osa-miR395v MIMAT0003876   Score: 4.5  TCONS_00100257 gene=XLOC_057032

Query:          1 GTGAAGTAT--T-TGGCGGAACTC 21
                  |||||:|||**|*|||*|:||||*
Sbjct:       1232 CACTTTATAGTACACCTCTTTGAT 1209
```

```
>osa-miR444a-3p.1 MIMAT0001617  Score: 4.0  TCONS_00021500 gene=XLOC_011874

Query:           1 TTGCTGCCT--CA-AGCTTGCTGC 21
                     |||||||||**||*||*||||:*|
Sbjct:         538 AACGACGGAGGGTGTCCAACGGGG 515


>osa-miR529b MIMAT0005015   Score: 4.5  TCONS_00098778 gene=XLOC_056041

Query:           1 AGAAGAGAG-A--GAGTACAGCTT 21
                     *|||:||||*|**|||:*||||||
Sbjct:          67 CCTTTTCTCCTCCCTCGAGTCGAA 44


>osa-miR1661-5p MIMAT0022871    Score: 4.0  TCONS_00044881 gene=XLOC_025454

Query:           1 GGATTGTTG--TC-TGGTTCAAGG 21
                     ||||||:||**||*||*||||*||
Sbjct:         137 CCTAACGACTGAGTACAAAGTCCC 114


>osa-miR1661-5p MIMAT0022871    Score: 4.5  TCONS_00088748 gene=XLOC_050158

Query:           1 GGATTGTTG-T--CTGGTTCAAGG 21
                     ||||:||||*:**||||||*|*|
Sbjct:         120 CCTAGCAACTGTTGACCAAGCTAC 97


>osa-miR395k MIMAT0000966   Score: 4.5  TCONS_00100257 gene=XLOC_057032

Query:           1 GTGAAGTGT--T-TGGGGGAACTC 21
                     |||||:|:|**|*|||:|:||||*
Sbjct:        1232 CACTTTATAGTACACCTCTTTGAT 1209


>osa-miR395d MIMAT0000960   Score: 4.5  TCONS_00100257 gene=XLOC_057032

Query:           1 GTGAAGTGT--T-TGGGGGAACTC 21
                     |||||:|:|**|*|||:|:||||*
Sbjct:        1232 CACTTTATAGTACACCTCTTTGAT 1209


>osa-miR2118q MIMAT0011756  Score: 4.5  TCONS_00044208 gene=XLOC_025107
```

```
Query:          1 TTCCCGATG-CC--TCCTATTCCTA 22
                  |||*|:||**||**|||||||||:|
Sbjct:       1124 AAGTGTTAGAGGATAGGATAAGGGT 1100
```

>osa-miR171h MIMAT0001077   Score: 4.5 TCONS_00015380 gene=XLOC_008313

```
Query:          1 GTGAGCCGA---ACCAATATCACT 21
                  ||||||*||***||*|||*|||||
Sbjct:        422 CACTCGCCTTCCTGTTTAGAGTGA 399
```

>osa-miR171h MIMAT0001077   Score: 4.5 TCONS_00015381 gene=XLOC_008313

```
Query:          1 GTGAGCCGA---ACCAATATCACT 21
                  ||||||*||***||*|||*|||||
Sbjct:        116 CACTCGCCTTCCTGTTTAGAGTGA 93
```

>osa-miR156k MIMAT0001020   Score: 4.0 TCONS_00043170 gene=XLOC_024537

```
Query:          1 TGACAGAAG--A-GAGAGAGCACA 21
                  ||||||||:**|*|||*||||||*
Sbjct:        719 ACTGTCTTT CTCCTCACTCGTGC 696
```

>osa-miR156k MIMAT0001020   Score: 4.0 TCONS_00043171 gene=XLOC_024537

```
Query:          1 TGACAGAAG--A-GAGAGAGCACA 21
                  ||||||||:**|*|||*||||||*
Sbjct:        719 ACTGTCTTT CTCCTCACTCGTGC 696
```

>osa-miR156k MIMAT0001020   Score: 4.0 TCONS_00043172 gene=XLOC_024537

```
Query:          1 TGACAGAAG--A-GAGAGAGCACA 21
                  ||||||||:**|*|||*||||||*
Sbjct:        719 ACTGTCTTT CTCCTCACTCGTGC 696
```

>osa-miR156k MIMAT0001020   Score: 4.0 TCONS_00043174 gene=XLOC_024537

```
Query:          1 TGACAGAAG--A-GAGAGAGCACA 21
```

```
                  ||||||||:**|*|||*|||||*
Sbjct:        719 ACTGTCTTT CTCCTCACTCGTGC 696


>osa-miR156k MIMAT0001020   Score: 4.0  TCONS_00043175 gene=XLOC_024537

Query:          1 TGACAGAAG--A-GAGAGAGCACA 21
                  ||||||||:**|*|||*|||||*
Sbjct:        719 ACTGTCTTT CTCCTCACTCGTGC 696


>osa-miR156k MIMAT0001020   Score: 4.0  TCONS_00043176 gene=XLOC_024537

Query:          1 TGACAGAAG--A-GAGAGAGCACA 21
                  ||||||||:**|*|||*|||||*
Sbjct:        719 ACTGTCTTT CTCCTCACTCGTGC 696


>osa-miR156k MIMAT0001020   Score: 4.5  TCONS_00068931 gene=XLOC_038677

Query:          1 TGACAGAAGA---GAGAGAGCACA 21
                  |||*||||||***||||||**||
Sbjct:        287 ACT TCTTCTAGCCTCTCTCCCGT 264


>osa-miR395j MIMAT0000965   Score: 4.5  TCONS_00100257 gene=XLOC_057032

Query:          1 GTGAAGTGT--T-TGGGGGAACTC 21
                  |||||:|:|**|*|||:|:||||*
Sbjct:       1232 CACTTTATAGTACACCTCTTTGAT 1209


>osa-miR160e-5p MIMAT0001030    Score: 2.5  TCONS_00111350 gene=XLOC_063639

Query:          1 TGCCTGGCT---CCCTGTATGCCG 21
                  |||||||||***||*|||||||||
Sbjct:        532 ACGGACCGAAAGGGAACATACGGC 509


>osa-miR171e-3p MIMAT0001066    Score: 4.5  TCONS_00068198 gene=XLOC_038327

Query:          1 TGATTGAGCC---GTGCCAATATC 21
                  ||||||||*|***||||*|:|:|
Sbjct:        201 ACTAACTCTGTTTCACGGGTGTGG 178
```

```
>osa-miR171e-3p MIMAT0001066    Score: 4.5  TCONS_00098418 gene=XLOC_055846

Query:          1 TGATTGAGCCG---TGCCAATATC 21
                  ||*||||:|*|***||||||:|||
Sbjct:        560 ACGAACTTGACTAAACGGTTGTAG 537


>osa-miR166h-3p MIMAT0001073    Score: 3.5  TCONS_00068571 gene=XLOC_038526

Query:          1 TCGGACCAG---GCTTCATTCCTC 21
                  ||||||||:***||||*|||||:|
Sbjct:        839 AGCCTGGTTAAGCGAACTAAGGGG 816


>osa-miR164f MIMAT0001089    Score: 4.5  TCONS_00100980 gene=XLOC_057427

Query:          1 TGGAGAAGC-A-G-GGCACGTGCA 21
                  ||||:||||*|*|*:||||::|*|
Sbjct:        732 ACCTTTTCGTTGCGTCGTGTGC T 709


>osa-miR156f-5p MIMAT0000623    Score: 2.0  TCONS_00043170 gene=XLOC_024537

Query:          1 TGACAGAAG--A-GAGTGAGCAC 20
                  ||||||||:**|*||||||||||
Sbjct:        719 ACTGTCTTT CTCCTCACTCGTG 697


>osa-miR156f-5p MIMAT0000623    Score: 2.0  TCONS_00043171 gene=XLOC_024537

Query:          1 TGACAGAAG--A-GAGTGAGCAC 20
                  ||||||||:**|*||||||||||
Sbjct:        719 ACTGTCTTT CTCCTCACTCGTG 697


>osa-miR156f-5p MIMAT0000623    Score: 2.0  TCONS_00043172 gene=XLOC_024537

Query:          1 TGACAGAAG--A-GAGTGAGCAC 20
                  ||||||||:**|*||||||||||
Sbjct:        719 ACTGTCTTT CTCCTCACTCGTG 697
```

```
>osa-miR156f-5p MIMAT0000623    Score: 2.0  TCONS_00043174 gene=XLOC_024537

Query:          1 TGACAGAAG--A-GAGTGAGCAC 20
                    ||||||||:**|*||||||||||
Sbjct:        719 ACTGTCTTT CTCCTCACTCGTG 697


>osa-miR156f-5p MIMAT0000623    Score: 2.0  TCONS_00043175 gene=XLOC_024537

Query:          1 TGACAGAAG--A-GAGTGAGCAC 20
                    ||||||||:**|*||||||||||
Sbjct:        719 ACTGTCTTT CTCCTCACTCGTG 697


>osa-miR156f-5p MIMAT0000623    Score: 2.0  TCONS_00043176 gene=XLOC_024537

Query:          1 TGACAGAAG--A-GAGTGAGCAC 20
                    ||||||||:**|*||||||||||
Sbjct:        719 ACTGTCTTT CTCCTCACTCGTG 697


>osa-miR156f-5p MIMAT0000623    Score: 4.5  TCONS_00076760 gene=XLOC_043053

Query:          1 TGACAGAAG-AG--AGTGAGCAC 20
                    ||*||||||*||**||||||*|*
Sbjct:        398 ACCGTCTTCCTCTTTCACTCCTT 376


>osa-miR156f-5p MIMAT0000623    Score: 4.5  TCONS_00087104 gene=XLOC_049223

Query:          1 TGACAGAAG-AG--AGTGAGCAC 20
                    ||||||||**||**|||:|:||*
Sbjct:       1646 ACTGTCTTACTCT TCATTTGTT 1624


>osa-miR160f-5p MIMAT0001031    Score: 4.5  TCONS_00111350 gene=XLOC_063639

Query:          1 TGCCTGGCT---CCCTGAATGCCA 21
                    |||||||||***||*||*|||||*
Sbjct:        532 ACGGACCGAAAGGGAACATACGGC 509


>osa-miR444d.1 MIMAT0005974 Score: 4.0  TCONS_00021500 gene=XLOC_011874
```

```
Query:          1 TTGCTGCCT--CA-AGCTTGCTGC 21
                  |||||||||**||*||*||||:*|
Sbjct:        538 AACGACGGAGGGTGTCCAACGGGG 515
```

>osa-miR166f MIMAT0000640   Score: 3.0 TCONS_00068571 gene=XLOC_038526

```
Query:          1 TCGGACCAG---GCTTCATTCCCC 21
                  ||||||||:***||||*|||||||
Sbjct:        839 AGCCTGGTTAAGCGAACTAAGGGG 816
```

>osa-miR319a-3p MIMAT0020915   Score: 3.5 TCONS_00066648 gene=XLOC_037329

```
Query:          1 ACTGGATGA---CGCGGGAGCTAA 21
                  |||||||||****||*||||||||
Sbjct:       2113 TGACCTACTTAATCGACCTCGATT 2090
```

>osa-miR395l MIMAT0000967   Score: 4.5 TCONS_00100257 gene=XLOC_057032

```
Query:          1 GTGAAGTGT--T-TGGGGGAACTC 21
                  |||||:|:|**|*|||:|:||||*
Sbjct:       1232 CACTTTATAGTACACCTCTTTGAT 1209
```

>osa-miR827 MIMAT0009981   Score: 4.5 TCONS_00069356 gene=XLOC_038879

```
Query:          1 TTAGATGAC-C--ATCAGCAAACA 21
                  |:|:|||||*|**||||||*||*|
Sbjct:       1794 AGTTTACTGTGTATAGTCG TTAT 1771
```

>osa-miR395s MIMAT0000968   Score: 4.5 TCONS_00100257 gene=XLOC_057032

```
Query:          1 GTGAAGTGT--T-TGGGGGAACTC 21
                  |||||:|:|**|*|||:|:||||*
Sbjct:       1232 CACTTTATAGTACACCTCTTTGAT 1209
```

>osa-miR395b MIMAT0000959   Score: 4.5 TCONS_00100257 gene=XLOC_057032

```
Query:          1 GTGAAGTGT--T-TGGGGGAACTC 21
                  |||||:|:|**|*|||:|:||||*
```

```
Sbjct:       1232 CACTTTATAGTACACCTCTTTGAT 1209
```

```
>osa-miR166i-5p MIMAT0022883    Score: 4.5  TCONS_00037124 gene=XLOC_021483

Query:          1 AATGCAGTT-T--GATCCAAGATC 21
                  ||::||*|:*|**|||||||:|||
Sbjct:        674 TTGTGTGAGTAGGCTAGGTTTTAG 651
```

```
>osa-miR166i-5p MIMAT0022883    Score: 4.5  TCONS_00037125 gene=XLOC_021483

Query:          1 AATGCAGTT-T--GATCCAAGATC 21
                  ||::||*|:*|**|||||||:|||
Sbjct:       2407 TTGTGTGAGTAGGCTAGGTTTTAG 2384
```

```
>osa-miR444c.1 MIMAT0005971 Score: 4.0  TCONS_00057276 gene=XLOC_031772

Query:          1 TGTTGTCTC--AA-GCTTGCTGCC 21
                  ||:|*||||**||**|||||||||
Sbjct:        140 ACGAAAGAGTTTTAGGAACGACGG 117
```

```
>osa-miR159a.2 MIMAT0009200 Score: 4.5  TCONS_00016968 gene=XLOC_009228

Query:          1 TTGCATGCC-C--CAGGAGCTGCA 21
                  |||||:||**|***||||:|||||
Sbjct:        738 AACGTGCGATGTCATCCTTGACGT 715
```

```
>osa-miR156c-5p MIMAT0000620    Score: 2.0  TCONS_00043170 gene=XLOC_024537

Query:          1 TGACAGAAG--A-GAGTGAGCAC 20
                  ||||||||:**|*||||||||||
Sbjct:        719 ACTGTCTTT CTCCTCACTCGTG 697
```

```
>osa-miR156c-5p MIMAT0000620    Score: 2.0  TCONS_00043171 gene=XLOC_024537

Query:          1 TGACAGAAG--A-GAGTGAGCAC 20
                  ||||||||:**|*||||||||||
Sbjct:        719 ACTGTCTTT CTCCTCACTCGTG 697
```

```
>osa-miR156c-5p MIMAT0000620    Score: 2.0  TCONS_00043172 gene=XLOC_024537

Query:          1 TGACAGAAG--A-GAGTGAGCAC 20
                  ||||||||:**|*||||||||||
Sbjct:        719 ACTGTCTTT CTCCTCACTCGTG 697


>osa-miR156c-5p MIMAT0000620    Score: 2.0  TCONS_00043174 gene=XLOC_024537

Query:          1 TGACAGAAG--A-GAGTGAGCAC 20
                  ||||||||:**|*||||||||||
Sbjct:        719 ACTGTCTTT CTCCTCACTCGTG 697


>osa-miR156c-5p MIMAT0000620    Score: 2.0  TCONS_00043175 gene=XLOC_024537

Query:          1 TGACAGAAG--A-GAGTGAGCAC 20
                  ||||||||:**|*||||||||||
Sbjct:        719 ACTGTCTTT CTCCTCACTCGTG 697


>osa-miR156c-5p MIMAT0000620    Score: 2.0  TCONS_00043176 gene=XLOC_024537

Query:          1 TGACAGAAG--A-GAGTGAGCAC 20
                  ||||||||:**|*||||||||||
Sbjct:        719 ACTGTCTTT CTCCTCACTCGTG 697


>osa-miR156c-5p MIMAT0000620    Score: 4.5  TCONS_00076760 gene=XLOC_043053

Query:          1 TGACAGAAG-AG--AGTGAGCAC 20
                  ||*||||||*||**||||||*|*
Sbjct:        398 ACCGTCTTCCTCTTTCACTCCTT 376


>osa-miR156c-5p MIMAT0000620    Score: 4.5  TCONS_00087104 gene=XLOC_049223

Query:          1 TGACAGAAG-AG--AGTGAGCAC 20
                  ||||||||**||**|||:|:||*
Sbjct:       1646 ACTGTCTTACTCT TCATTTGTT 1624


>osa-miR166e-5p MIMAT0022859    Score: 4.5  TCONS_00072868 gene=XLOC_040881
```

```
Query:           1 GGAATGTTG-T--CTGGTTCAAGG 21
                   |:|||||||*|**|||||*|*|:|
Sbjct:        2966 CTTTACAACAATCGACCACGGTTC 2943


>osa-miR166e-5p MIMAT0022859    Score: 4.5  TCONS_00072869 gene=XLOC_040881

Query:           1 GGAATGTTG-T--CTGGTTCAAGG 21
                   |:|||||||*|**|||||*|*|:|
Sbjct:        3719 CTTTACAACAATCGACCACGGTTC 3696


>osa-miR166e-5p MIMAT0022859    Score: 4.5  TCONS_00102306 gene=XLOC_058088

Query:           1 GGAATGTTGT--C-TGGTTCAAGG 21
                   |||||||::|**|*||||*|||::
Sbjct:         452 CCTTACAGTACTGTACCACGTTTT 429


>osa-miR168a-5p MIMAT0001045    Score: 4.5  TCONS_00018461 gene=XLOC_010132

Query:           1 TCGCTTGGT---GCAGATCGGGAC 21
                   :||*|||||***:||:|:||||||
Sbjct:        1113 GGCCAACCATTGTGTTTGGCCCTG 1090


>osa-miR168a-5p MIMAT0001045    Score: 4.5  TCONS_00018462 gene=XLOC_010132

Query:           1 TCGCTTGGT---GCAGATCGGGAC 21
                   :||*|||||***:||:|:||||||
Sbjct:        1160 GGCCAACCATTGTGTTTGGCCCTG 1137


>osa-miR168a-5p MIMAT0001045    Score: 4.5  TCONS_00069605 gene=XLOC_038990

Query:           1 TCGCTTGGT--GC-AGATCGGGAC 21
                   *|||||||***||*|||||*||||
Sbjct:         464 CGCGAACCCGCCGATCTAGGCCTG 441


>osa-miR168a-5p MIMAT0001045    Score: 3.5  TCONS_00087860 gene=XLOC_049723

Query:           1 TCGCTTGGT-G-C-AGATCGGGAC 21
```

```
                    |*|||||||*|*|*|||||:|:||
Sbjct:       4770 ATCGAACCAACGG TCTAGTCTTG 4747


>osa-miR168a-5p MIMAT0001045    Score: 3.5  TCONS_00087861 gene=XLOC_049723

Query:          1 TCGCTTGGT--G-CAGATCGGGAC 21
                    |*|||||||**|*||||||:|:||
Sbjct:       4909 ATCGAACCAA CGGTCTAGTCTTG 4886


>osa-miR156i MIMAT0000626    Score: 2.0  TCONS_00043170 gene=XLOC_024537

Query:          1 TGACAGAAG--A-GAGTGAGCAC 20
                    ||||||||:**|*||||||||||
Sbjct:        719 ACTGTCTTT CTCCTCACTCGTG 697


>osa-miR156i MIMAT0000626    Score: 2.0  TCONS_00043171 gene=XLOC_024537

Query:          1 TGACAGAAG--A-GAGTGAGCAC 20
                    ||||||||:**|*||||||||||
Sbjct:        719 ACTGTCTTT CTCCTCACTCGTG 697


>osa-miR156i MIMAT0000626    Score: 2.0  TCONS_00043172 gene=XLOC_024537

Query:          1 TGACAGAAG--A-GAGTGAGCAC 20
                    ||||||||:**|*||||||||||
Sbjct:        719 ACTGTCTTT CTCCTCACTCGTG 697


>osa-miR156i MIMAT0000626    Score: 2.0  TCONS_00043174 gene=XLOC_024537

Query:          1 TGACAGAAG--A-GAGTGAGCAC 20
                    ||||||||:**|*||||||||||
Sbjct:        719 ACTGTCTTT CTCCTCACTCGTG 697


>osa-miR156i MIMAT0000626    Score: 2.0  TCONS_00043175 gene=XLOC_024537

Query:          1 TGACAGAAG--A-GAGTGAGCAC 20
                    ||||||||:**|*||||||||||
Sbjct:        719 ACTGTCTTT CTCCTCACTCGTG 697
```

```
>osa-miR156i MIMAT0000626   Score: 2.0  TCONS_00043176 gene=XLOC_024537

Query:          1 TGACAGAAG--A-GAGTGAGCAC 20
                  ||||||||:**|*||||||||||
Sbjct:        719 ACTGTCTTT CTCCTCACTCGTG 697


>osa-miR156i MIMAT0000626   Score: 4.5  TCONS_00076760 gene=XLOC_043053

Query:          1 TGACAGAAG-AG--AGTGAGCAC 20
                  ||*||||||*||**||||||*|*
Sbjct:        398 ACCGTCTTCCTCTTTCACTCCTT 376


>osa-miR156i MIMAT0000626   Score: 4.5  TCONS_00087104 gene=XLOC_049223

Query:          1 TGACAGAAG-AG--AGTGAGCAC 20
                  ||||||||**||**|||:|:||*
Sbjct:       1646 ACTGTCTTACTCT TCATTTGTT 1624


>osa-miR171c-3p MIMAT0001064    Score: 4.5  TCONS_00068198 gene=XLOC_038327

Query:          1 TGATTGAGCC---GTGCCAATATC 21
                  ||||||||*|***|||||*|:|:|
Sbjct:        201 ACTAACTCTGTTTCACGGGTGTGG 178


>osa-miR171c-3p MIMAT0001064    Score: 4.5  TCONS_00098418 gene=XLOC_055846

Query:          1 TGATTGAGCCG---TGCCAATATC 21
                  ||*||||:|*|***||||||:|||
Sbjct:        560 ACGAACTTGACTAAACGGTTGTAG 537


>osa-miR395t MIMAT0000969   Score: 4.0  TCONS_00100257 gene=XLOC_057032

Query:          1 GTGAAGTGT--T-TGGGGAAACTC 21
                  |||||:|:|**|*|||:||||||*
Sbjct:       1232 CACTTTATAGTACACCTCTTTGAT 1209
```

```
>osa-miR395n MIMAT0003871   Score: 4.5  TCONS_00100257 gene=XLOC_057032

Query:          1 GTGAAGTGT--T-TGGGGGAACTC 21
                  |||||:|:|**|*|||:|:||||*
Sbjct:       1232 CACTTTATAGTACACCTCTTTGAT 1209


>osa-miR160d-5p MIMAT0000631    Score: 3.5  TCONS_00111350 gene=XLOC_063639

Query:          1 TGCCTGGCT---CCCTGTATGCCA 21
                  |||||||||***||*||||||||*
Sbjct:        532 ACGGACCGAAAGGGAACATACGGC 509


>osa-miR408-3p MIMAT0001079 Score: 4.0  TCONS_00056472 gene=XLOC_031355

Query:          1 CTGCACTGCCT---CTTCCCTGGC 21
                  ||||||*||||***||||||:*||
Sbjct:        259 GACGTGCCGGACCGGAAGGGGGCG 236


>osa-miR408-3p MIMAT0001079 Score: 4.5  TCONS_00051144 gene=XLOC_028659

Query:          1 CTGCACTGC-CT--CTTCCCTGGC 21
                  |:||*|:||*|:**||||||:|||
Sbjct:        172 GGCGAGGCGTGGTCGAAGGGGCCG 149


>osa-miR408-3p MIMAT0001079 Score: 4.5  TCONS_00094855 gene=XLOC_053743

Query:          1 CTGCACTGC--CT-CTTCCCTGGC 21
                  ||||*||||**||**||||||*||
Sbjct:        699 GACGAGACGTAGACTAAGGGAACG 676


>osa-miR171b MIMAT0001063   Score: 4.5  TCONS_00068198 gene=XLOC_038327

Query:          1 TGATTGAGCC---GTGCCAATATC 21
                  ||||||||*|***|||||*|:|:|
Sbjct:        201 ACTAACTCTGTTTCACGGGTGTGG 178


>osa-miR171b MIMAT0001063   Score: 4.5  TCONS_00098418 gene=XLOC_055846
```

```
Query:          1 TGATTGAGCCG---TGCCAATATC 21
                  ||*||||:|*|***||||||:|||
Sbjct:        560 ACGAACTTGACTAAACGGTTGTAG 537
```

>osa-miR171f-3p MIMAT0001067   Score: 4.5 TCONS_00068198 gene=XLOC_038327

```
Query:          1 TGATTGAGCC---GTGCCAATATC 21
                  ||||||||*|***|||||*|:|:|
Sbjct:        201 ACTAACTCTGTTTCACGGGTGTGG 178
```

>osa-miR171f-3p MIMAT0001067   Score: 4.5 TCONS_00098418 gene=XLOC_055846

```
Query:          1 TGATTGAGCCG---TGCCAATATC 21
                  ||*||||:|*|***||||||:|||
Sbjct:        560 ACGAACTTGACTAAACGGTTGTAG 537
```

>osa-miR166l-3p MIMAT0001038   Score: 4.5 TCONS_00011308 gene=XLOC_006054

```
Query:          1 TCGGACCAG---GCTTCAATCCCT 21
                  ||||||||****||:|||||*||:
Sbjct:        462 AGCCTGGTACTGCGGAGTTAAGGG 439
```

>osa-miR166l-3p MIMAT0001038   Score: 4.5 TCONS_00068571 gene=XLOC_038526

```
Query:          1 TCGGACCAG---GCTTCAATCCCT 21
                  ||||||||:***||||*|*||||:
Sbjct:        839 AGCCTGGTTAAGCGAACTAAGGGG 816
```

>osa-miR166a-5p MIMAT0022855   Score: 4.5 TCONS_00072868 gene=XLOC_040881

```
Query:          1 GGAATGTTG-T--CTGGTTCAAGG 21
                  |:|||||||*|**|||||*|*|:|
Sbjct:       2966 CTTTACAACAATCGACCACGGTTC 2943
```

>osa-miR166a-5p MIMAT0022855   Score: 4.5 TCONS_00072869 gene=XLOC_040881

```
Query:          1 GGAATGTTG-T--CTGGTTCAAGG 21
                  |:|||||||*|**|||||*|*|:|
```

```
Sbjct:       3719 CTTTACAACAATCGACCACGGTTC 3696


>osa-miR166a-5p MIMAT0022855    Score: 4.5  TCONS_00102306 gene=XLOC_058088

Query:          1 GGAATGTTGT--C-TGGTTCAAGG 21
                  |||||||::|**|*||||*|||::
Sbjct:        452 CCTTACAGTACTGTACCACGTTTT 429


>osa-miR156b-3p MIMAT0022845    Score: 4.0  TCONS_00107603 gene=XLOC_061457

Query:          1 GCTCACTCT---CTATCTGTCAGC 21
                  |||||||||***||*||*|:||||
Sbjct:        418 CGAGTGAGAGAAGACAGTCGGTCG 395


>osa-miR156b-3p MIMAT0022845    Score: 4.0  TCONS_00107604 gene=XLOC_061457

Query:          1 GCTCACTCT---CTATCTGTCAGC 21
                  |||||||||***||*||*|:||||
Sbjct:        399 CGAGTGAGAGAAGACAGTCGGTCG 376


>osa-miR164e MIMAT0001035   Score: 4.0  TCONS_00055782 gene=XLOC_031038

Query:          1 TGGAGAAGC--AG-GGCACGTGAG 21
                  |||||||||**||*|||**|:|||
Sbjct:        547 ACCTCTTCGAGTCGCCGCCCGCTC 524


>osa-miR164e MIMAT0001035   Score: 4.0  TCONS_00055783 gene=XLOC_031038

Query:          1 TGGAGAAGC--AG-GGCACGTGAG 21
                  |||||||||**||*|||**|:|||
Sbjct:        547 ACCTCTTCGAGTCGCCGCCCGCTC 524


>osa-miR164e MIMAT0001035   Score: 4.0  TCONS_00055784 gene=XLOC_031038

Query:          1 TGGAGAAGC--AG-GGCACGTGAG 21
                  |||||||||**||*|||**|:|||
Sbjct:        553 ACCTCTTCGAGTCGCCGCCCGCTC 530
```

```
>osa-miR164e MIMAT0001035   Score: 4.0  TCONS_00055785 gene=XLOC_031038

Query:          1 TGGAGAAGC--AG-GGCACGTGAG 21
                    |||||||||**||*|||**|:|||
Sbjct:        553 ACCTCTTCGAGTCGCCGCCCGCTC 530


>osa-miR166e-3p MIMAT0000639    Score: 4.0  TCONS_00068571 gene=XLOC_038526

Query:          1 TCGAACCAG---GCTTCATTCCCC 21
                    |||*||||:***||||*|||||||
Sbjct:        839 AGCCTGGTTAAGCGAACTAAGGGG 816


>osa-miR166j-3p MIMAT0001088    Score: 3.0  TCONS_00068571 gene=XLOC_038526

Query:          1 TCGGACCAG---GCTTCATTCCCC 21
                    ||||||||:***||||*|||||||
Sbjct:        839 AGCCTGGTTAAGCGAACTAAGGGG 816


>osa-miR1435 MIMAT0005986   Score: 3.5  TCONS_00010861 gene=XLOC_005835

Query:          1 TTTCTTAAGT--C-AAACTTTTT 20
                    |||*|:||||**|*||||:||||
Sbjct:        418 AAACAGTTCACGGTTTTGGAAAA 396


>osa-miR1435 MIMAT0005986   Score: 4.5  TCONS_00014791 gene=XLOC_007899

Query:          1 TTTCTTAAG--T-CAAACTTTTT 20
                    |||*|**||**|*||||||||||
Sbjct:         74 AAA ACATCACAGGTTTGAAAAA 52


>osa-miR1435 MIMAT0005986   Score: 4.5  TCONS_00029525 gene=XLOC_016842

Query:          1 TTTCTTAAG---TCAAACTTTTT 20
                    ||||||*||***||||||*|*||
Sbjct:        203 AAAGAAATCACTAGTTTGTACAA 181


>osa-miR1435 MIMAT0005986   Score: 4.0  TCONS_00034465 gene=XLOC_019858
```

```
Query:           1 TTTCTTAAG--T-CAAACTTTTT 20
                   ||||::||:**|*|*||||||||
Sbjct:         935 AAAGGGTTTGAA GGTTGAAAAA 913


>osa-miR1435 MIMAT0005986   Score: 4.5  TCONS_00071735 gene=XLOC_040137

Query:           1 TTTCTTAAG---TCAAACTTTTT 20
                   ||||||*|:***||*|||||||:
Sbjct:         628 AAAGAAGTTTAAAGGTTGAAAAG 606


>osa-miR1435 MIMAT0005986   Score: 4.5  TCONS_00090532 gene=XLOC_051185

Query:           1 TTTCTTAAG-T--CAAACTTTTT 20
                   |||*||*||*|**|||||||*||
Sbjct:        1148 AAACAACTCAAG GTTTGAACAA 1126


>osa-miR1435 MIMAT0005986   Score: 4.0  TCONS_00104489 gene=XLOC_059506

Query:           1 TTTCTTAAGT---CAAACTTTTT 20
                   ||||||||||***|||*||:|*|
Sbjct:         271 AAAGAATTCAGTCGTTGGAGACA 249


>osa-miR1435 MIMAT0005986   Score: 4.0  TCONS_00104492 gene=XLOC_059506

Query:           1 TTTCTTAAGT---CAAACTTTTT 20
                   ||||||||||***|||*||:|*|
Sbjct:         250 AAAGAATTCAGTCGTTGGAGACA 228


>osa-miR1435 MIMAT0005986   Score: 4.0  TCONS_00110274 gene=XLOC_062969

Query:           1 TTTCTTAAG---TCAAACTTTTT 20
                   ||||||*|:***||*||||||||
Sbjct:        1417 AAAGAAGTTTGAAGGTTGAAAAA 1395


>osa-miR1435 MIMAT0005986   Score: 4.5  TCONS_00107680 gene=XLOC_061504

Query:           1 TTTCTTAAG---TCAAACTTTTT 20
```

```
                  ||||||*|:***||*|||||||:
Sbjct:        317 AAAGAAGTTTGAAGGTTGAAAAG 295


>osa-miR444b.1 MIMAT0005969 Score: 4.0  TCONS_00057276 gene=XLOC_031772

Query:          1 TGTTGTCTC--AA-GCTTGCTGCC 21
                  ||:|*||||**||**|||||||||
Sbjct:        140 ACGAAAGAGTTTTAGGAACGACGG 117


>osa-miR395c MIMAT0000972   Score: 4.5  TCONS_00029176 gene=XLOC_016647

Query:          1 GTGAAGTGT---TTGGAGGAACTC 21
                  ||*||||*|***:||:||||||||
Sbjct:        481 CAATTCAAATCGGACTTCCTTGAG 458


>osa-miR395c MIMAT0000972   Score: 4.0  TCONS_00100257 gene=XLOC_057032

Query:          1 GTGAAGTGT--T-TGGAGGAACTC 21
                  |||||:|:|**|*||||:||||*
Sbjct:       1232 CACTTTATAGTACACCTCTTTGAT 1209


>osa-miR156a MIMAT0000618   Score: 2.0  TCONS_00043170 gene=XLOC_024537

Query:          1 TGACAGAAG--A-GAGTGAGCAC 20
                  ||||||||:**|*||||||||||
Sbjct:        719 ACTGTCTTT CTCCTCACTCGTG 697


>osa-miR156a MIMAT0000618   Score: 2.0  TCONS_00043171 gene=XLOC_024537

Query:          1 TGACAGAAG--A-GAGTGAGCAC 20
                  ||||||||:**|*||||||||||
Sbjct:        719 ACTGTCTTT CTCCTCACTCGTG 697


>osa-miR156a MIMAT0000618   Score: 2.0  TCONS_00043172 gene=XLOC_024537

Query:          1 TGACAGAAG--A-GAGTGAGCAC 20
                  ||||||||:**|*||||||||||
Sbjct:        719 ACTGTCTTT CTCCTCACTCGTG 697
```

```
>osa-miR156a MIMAT0000618   Score: 2.0  TCONS_00043174 gene=XLOC_024537

Query:          1 TGACAGAAG--A-GAGTGAGCAC 20
                  ||||||||:**|*||||||||||
Sbjct:        719 ACTGTCTTT CTCCTCACTCGTG 697


>osa-miR156a MIMAT0000618   Score: 2.0  TCONS_00043175 gene=XLOC_024537

Query:          1 TGACAGAAG--A-GAGTGAGCAC 20
                  ||||||||:**|*||||||||||
Sbjct:        719 ACTGTCTTT CTCCTCACTCGTG 697


>osa-miR156a MIMAT0000618   Score: 2.0  TCONS_00043176 gene=XLOC_024537

Query:          1 TGACAGAAG--A-GAGTGAGCAC 20
                  ||||||||:**|*||||||||||
Sbjct:        719 ACTGTCTTT CTCCTCACTCGTG 697


>osa-miR156a MIMAT0000618   Score: 4.5  TCONS_00076760 gene=XLOC_043053

Query:          1 TGACAGAAG-AG--AGTGAGCAC 20
                  ||*||||||*||**||||||*|*
Sbjct:        398 ACCGTCTTCCTCTTTCACTCCTT 376


>osa-miR156a MIMAT0000618   Score: 4.5  TCONS_00087104 gene=XLOC_049223

Query:          1 TGACAGAAG-AG--AGTGAGCAC 20
                  ||||||||**||**|||:|:||*
Sbjct:       1646 ACTGTCTTACTCT TCATTTGTT 1624


>osa-miR166k-5p MIMAT0022870    Score: 4.5  TCONS_00010856 gene=XLOC_005835

Query:          1 GGTTTGTTG---TCTGGCTCGAGG 21
                  :||||||||***|||||*|*|||:
Sbjct:       1685 TCAAACAACAGTAGACCAATCTCT 1662
```

```
>osa-miR166k-5p MIMAT0022870    Score: 4.5  TCONS_00027308 gene=XLOC_015481

Query:          1 GGTTTGTTGT-C--TGGCTCGAGG 21
                  ||||:|:||:*|**:*||||||||
Sbjct:        209 CCAAGCGACGTGTCGGCGAGCTCC 186


>osa-miR156h-5p MIMAT0031156    Score: 2.0  TCONS_00043170 gene=XLOC_024537

Query:          1 TGACAGAAG--A-GAGTGAGCAC 20
                  ||||||||:**|*||||||||||
Sbjct:        719 ACTGTCTTT CTCCTCACTCGTG 697


>osa-miR156h-5p MIMAT0031156    Score: 2.0  TCONS_00043171 gene=XLOC_024537

Query:          1 TGACAGAAG--A-GAGTGAGCAC 20
                  ||||||||:**|*||||||||||
Sbjct:        719 ACTGTCTTT CTCCTCACTCGTG 697


>osa-miR156h-5p MIMAT0031156    Score: 2.0  TCONS_00043172 gene=XLOC_024537

Query:          1 TGACAGAAG--A-GAGTGAGCAC 20
                  ||||||||:**|*||||||||||
Sbjct:        719 ACTGTCTTT CTCCTCACTCGTG 697


>osa-miR156h-5p MIMAT0031156    Score: 2.0  TCONS_00043174 gene=XLOC_024537

Query:          1 TGACAGAAG--A-GAGTGAGCAC 20
                  ||||||||:**|*||||||||||
Sbjct:        719 ACTGTCTTT CTCCTCACTCGTG 697


>osa-miR156h-5p MIMAT0031156    Score: 2.0  TCONS_00043175 gene=XLOC_024537

Query:          1 TGACAGAAG--A-GAGTGAGCAC 20
                  ||||||||:**|*||||||||||
Sbjct:        719 ACTGTCTTT CTCCTCACTCGTG 697


>osa-miR156h-5p MIMAT0031156    Score: 2.0  TCONS_00043176 gene=XLOC_024537
```

Query: 1 TGACAGAAG--A-GAGTGAGCAC 20
||||||||:**|*||||||||||
Sbjct: 719 ACTGTCTTT CTCCTCACTCGTG 697

>osa-miR156h-5p MIMAT0031156 Score: 4.5 TCONS_00076760 gene=XLOC_043053

Query: 1 TGACAGAAG-AG--AGTGAGCAC 20
||*||||||*||**||||||*|*
Sbjct: 398 ACCGTCTTCCTCTTTCACTCCTT 376

>osa-miR156h-5p MIMAT0031156 Score: 4.5 TCONS_00087104 gene=XLOC_049223

Query: 1 TGACAGAAG-AG--AGTGAGCAC 20
||||||||**||**|||:|:||*
Sbjct: 1646 ACTGTCTTACTCT TCATTTGTT 1624

>osa-miR171d-3p MIMAT0001065 Score: 4.5 TCONS_00068198 gene=XLOC_038327

Query: 1 TGATTGAGCC---GTGCCAATATC 21
||||||||*|***|||||*|:|:|
Sbjct: 201 ACTAACTCTGTTTCACGGGTGTGG 178

>osa-miR171d-3p MIMAT0001065 Score: 4.5 TCONS_00098418 gene=XLOC_055846

Query: 1 TGATTGAGCCG---TGCCAATATC 21
||*||||:|*|***||||||:|||
Sbjct: 560 ACGAACTTGACTAAACGGTTGTAG 537

>osa-miR397b MIMAT0000981 Score: 4.5 TCONS_00037671 gene=XLOC_021753

Query: 1 TTATTGAGT--GC-AGCGTTGATG 21
||*||||*|**||*|||||||*||
Sbjct: 2975 AACAACTAAGTCGATCGCAACCAC 2952

>osa-miR166c-3p MIMAT0000637 Score: 3.0 TCONS_00068571 gene=XLOC_038526

Query: 1 TCGGACCAG---GCTTCATTCCCC 21
||||||||:***||||*|||||||

```
Sbjct:        839 AGCCTGGTTAAGCGAACTAAGGGG 816

>osa-miR164a MIMAT0000634   Score: 4.5  TCONS_00013466 gene=XLOC_007072

Query:          1 TGGAGAAGCA---GGGCACGTGCA 21
                  ||:|||||||:::*|||::| ||:
Sbjct:        453 ACGTCTTCGTAAGTCCGTGC-CGC 430

>osa-miR164b MIMAT0000634   Score: 4.5  TCONS_00013466 gene=XLOC_007072

Query:          1 TGGAGAAGCA---GGGCACGTGCA 21
                  ||:|||||||:::*|||::| ||:
Sbjct:        453 ACGTCTTCGTAAGTCCGTGC-CGC 430

>osa-miR164f MIMAT0000634   Score: 4.5  TCONS_00013466 gene=XLOC_007072

Query:          1 TGGAGAAGCA---GGGCACGTGCA 21
                  ||:|||||||:::*|||::| ||:
Sbjct:        453 ACGTCTTCGTAAGTCCGTGC-CGC 430
```
